# The ORF8 protein of SARS-CoV-2 mediates immune evasion through down-regulating MHC-Ι

**DOI:** 10.1073/pnas.2024202118

**Published:** 2021-05-21

**Authors:** Yiwen Zhang, Yingshi Chen, Yuzhuang Li, Feng Huang, Baohong Luo, Yaochang Yuan, Baijin Xia, Xiancai Ma, Tao Yang, Fei Yu, Jun Liu, Bingfeng Liu, Zheng Song, Jingliang Chen, Shumei Yan, Liyang Wu, Ting Pan, Xu Zhang, Rong Li, Wenjing Huang, Xin He, Fei Xiao, Junsong Zhang, Hui Zhang

**Affiliations:** ^a^Institute of Human Virology, Key Laboratory of Tropical Disease Control of Ministry of Education, Guangdong Engineering Research Center for Antimicrobial Agent and Immunotechnology, Zhongshan School of Medicine, Sun Yat-sen University, 510080, Guangzhou, Guangdong, China;; ^b^Department of Respiratory Diseases, Guangzhou Women and Children Hospital, 510010, Guangzhou, Guangdong, China;; ^c^Guangdong Provincial People’s Hospital, Guangdong Academy of Medical Sciences, 510000, Guangzhou, Guangdong, China;; ^d^Department of Infectious Diseases, The Fifth Affiliated Hospital, Sun Yat-sen University, 519000, Zhuhai, Guangdong, China

**Keywords:** SARS-CoV-2, immune evasion, MHC-Ι, ORF8

## Abstract

COVID-19, caused by severe acute respiratory syndrome coronavirus 2 (SARS-CoV-2), has become a global pandemic and has claimed over 2 million lives worldwide. Although the genetic sequences of SARS-CoV and SARS-CoV-2 have high homology, the clinical and pathological characteristics of COVID-19 differ significantly from those of SARS. How and whether SARS-CoV-2 evades (cellular) immune surveillance requires further elucidation. In this study, we show that SARS-CoV-2 infection leads to major histocompability complex class Ι (MHC-Ι) down-regulation both in vitro and in vivo. The viral protein encoded by open reading frame 8 (ORF8) of SARS-CoV-2, which shares the least homology with SARS-CoV among all viral proteins, directly interacts with MHC-Ι molecules and mediates their down-regulation. In ORF8-expressing cells, MHC-Ι molecules are selectively targeted for lysosomal degradation via autophagy. Thus, SARS-CoV-2–infected cells are much less sensitive to lysis by cytotoxic T lymphocytes. Because ORF8 protein impairs the antigen presentation system, inhibition of ORF8 could be a strategy to improve immune surveillance.

## Significance

We report that SARS-CoV-2 utilizes its ORF8 protein as a unique mechanism to alter the expression of surface MHC-Ι expression to evade immune surveillance. Our study is significant for providing an understanding of the pathogenesis of SARS-CoV-2 and will provide additional perspective to the intensive ongoing investigation into the mechanism and function of T cell antiviral immunity in COVID-19.

Since the outbreak of COVID-19, the disease has been spreading worldwide rapidly (1–4). Although both COVID-19 and severe acute respiratory syndrome (SARS) cause severe respiratory illness, epidemiological and clinical data suggest that the disease spectrum of COVID-19 is markedly different from that of SARS. COVID-19 shows a longer incubation period (around 6.4 d, range: 0 to 24 d) than SARS; interpersonal transmission could occur from presymptomatic individuals (5, 6); asymptomatic infection has been widely reported and severely jeopardizes the prevention system in a community (5); a significant portion of recovered patients still shed genetic materials of the virus in the upper respiratory tract and digestive tract, leading to their hospitalization for a considerably longer time (7–9); and some recovered patients show redetectable viral RNA after being discharged from the hospital (7). The desynchronization of viral titer and clinical symptom development suggest that SARS coronavirus 2 (SARS-CoV-2) could have undergone extensive replication in infected host cells without being effectively detected by host antiviral immunity (10).

Cytotoxic T lymphocytes (CTLs) play an important role in controlling viral infection by directly eradicating the virus-infected cells (11). In a virus-infected cell, major histocompability complex class Ι (MHC-Ι) molecules present peptides derived from various viral proteins. Once the T cell receptor on CD8^+^ T cells recognizes the special signal presented by MHC-Ι–peptide complex, CTLs release various toxic substances (i.e., perforins, granzyme, and FasL) that directly induce the death of viral-infected cells as well as cytokines such as interferon-γ, TNF-α, and IL-2 (11). Thus, the cells supporting viral replication will be eliminated, and the spread of viruses will be effectively prevented (12). Some viruses that cause chronic infection, such as HIV type 1 (HIV-1) and Kaposi’s sarcoma–associated herpes virus (KSHV), can disrupt antigen presentation for immune activation by down-regulating MHC-Ι expression on the surface of cells and evading immune surveillance (13–15). In the current study, we investigated whether the SARS-CoV-2 virus could affect the antigen presentation system and assist viruses in evading immune surveillance.

In this study, we report that SARS-CoV-2 virus leads to MHC-Ι down-regulation in both infected human angiotensin–converting enzyme 2 (hACE2)-expressing HEK293T (HEK293T/Hace2) cells and infected lung epithelial cells of hACE2 transgene mice. We screened all SARS-CoV-2 structural proteins and unidentified open reading frames (ORFs) and found that ORF8, which shares the least homology with SARS-CoV among all viral proteins, can directly interact with MHC-Ι molecules and mediate their down-regulation through the autophagy pathway. In addition, we obtained healthy human donor–derived CTLs sensitized to the SARS-CoV-2 epitope SARS-CoV spike protein–derived peptide-1 (SSp-1, RLNEVAKNL) and CTLs isolated from a patient recovering from COVID-19 that responded to a mixture of SARS-CoV-2 peptides. ORF8-expressing cells and SARS-CoV-2–infected cells were found to be more resistant to CTL lysis. Knockdown of ORF8 protein expression in SARS-CoV-2–infected cells restored MHC-Ι expression and consequently cell sensitivity to CTL lysis. Collectively, our results strongly suggested that ORF8 induced MHC-Ι down-regulation and provided protection against CTLs in SARS-CoV-2–infected host cells.

## Results

### SARS-CoV-2 Infection Leads to MHC-Ι Down-Regulation through ORF8.

**Fig. 1.**
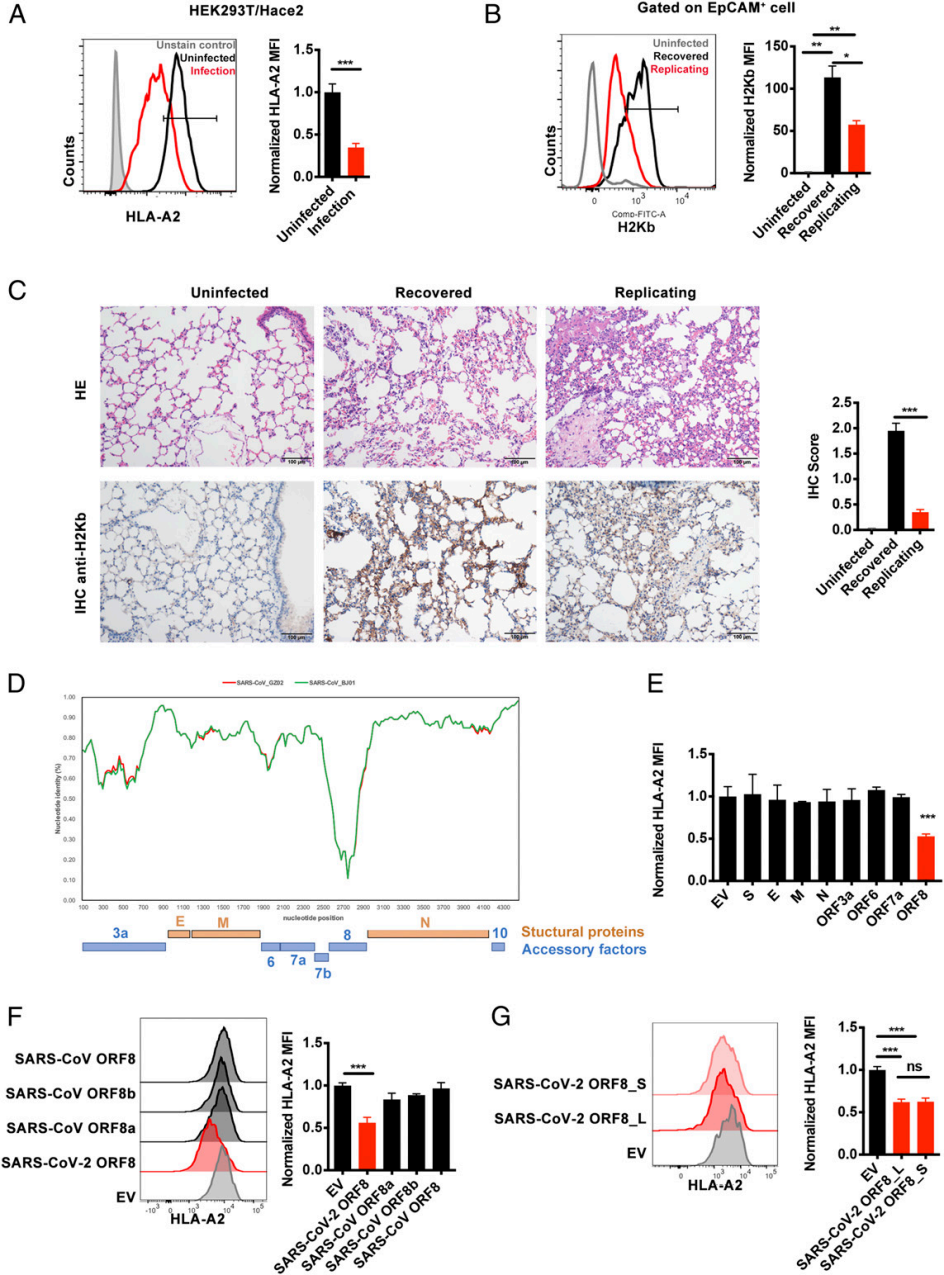
SARS-CoV-2 infection leads to MHC-Ι down-regulation through ORF8. (*A*) The ACE2-expressing HEK293T cells (HEK293T/Hace2) were infected with SARS-CoV-2 (hCoV-19/CHN/SYSU-IHV/2020) (MOI = 1). At 48 h after infection, cells were collected for flow cytometry analysis (*n* = 6). Mean fluorescence intensity (MFI) was normalized to uninfected group. (*B*) hACE2 mice were intranasally infected with 4 × 10^3^ PFU (recovered), 4 × 10^4^ PFU (replicating) SARS-CoV-2 virus, or uninfected as control. At day 6 after infection, total suspended cells of the lung tissue were collected for flow cytometry analysis. MFI of H2Kb^+^ cells (gated on EpCAM^+^ cells) were shown (*n* = 3). MFI was normalized to uninfected group. (*C*) Hematoxylin and eosin staining and immunohistochemistry against H2Kb were evaluated in lungs of infected mice in *B*. (*D*) Similarity plot based on the genome sequence of SARS-CoV-2_WHU01 (accession no. MN988668) and the genome sequences of SARS-CoV_BJ01 (AY278488) and SARS-CoV_GZ02 (AY390556) were used as reference sequences. The nucleotide position started from the orf3a gene of SARS-CoV-2. (*E*–*G*) The effect of different viral proteins on the expression of HLA-A2. The viral protein–expressing plasmids were transfected into HEK293T cell line, and cells were collected at 48 h after transfection for flow cytometry analysis to analyze the MFI of HLA-A2^+^ cells (*n* = 5) normalized to empty vector (EV) group. The plasmids expressing SARS-CoV-2 structural proteins and ORFs (*E*), SARS-CoV ORF8, ORF8b and ORF8a (*F*), and L and S subtype of SARS-CoV-2 ORF8 or EV (*G*) were used. The data were shown as mean ± SD (error bars). Student’s *t* test and one-way ANOVA was used. *P* < 0.05 indicates a statistically significance difference; *P < 0.05; **P < 0.01; ***P < 0.001.

Pathogenic biological events such as viral infection and tumorigenesis often take advantage of their ability in manipulating the antigen presentation system to evade immune surveillance (12). Therefore, we tested the hypothesis that SARS-CoV-2 might impair antigen presentation. An authentic SARS-CoV-2 strain, isolated in-house from a patient with COVID-19 and named hCoV-19/CHN/SYSU-IHV/2020 (16, 17), was used to infect HEK293T/Hace2 cells at a multiplicity of infection (MOI) of 1.0. As shown in Fig. 1*A*, the surface expression of MHC-1 in the infected cells decreased significantly. To further investigate the in vivo pathology of this phenomenon, we infected hACE2 mice with SARS-CoV-2, as we previously described (16). The hACE2 mice were divided into three groups, control (uninfected), inoculated intranasally with 4 × 10^3^ PFU, or 4 × 10^4^ PFU of SARS-CoV-2 virus. At day 6 after infection, viral RNAs were detected with quantitative RT-PCR. Viruses were found to be actively replicating in the 4 × 10^4^ PFU (replicating group), indicated by the presence of more than 10,000 copies of viral RNA. At the same time, mice from the 4 × 10^3^ PFU group recovered from the virus (recovered group), indicated by the absence of viral RNA. Then, lung tissues were obtained from the mice, and the MHC-Ι expression of lung epithelial cells, one of the host cells infected by SARS-CoV-2, were further analyzed by flow cytometry (*SI Appendix*, Fig. S1*A*) (18, 19). In line with previous reports that MHC-Ι expression was extremely low on the lung epithelial cells and up-regulated upon virus infection, we also have observed an up-regulation of MHC-Ι after SARS-CoV-2 infection (Fig. 1*B*), possibly the consequence of innate antiviral inflammatory response (20, 21). However, the MHC-Ι expression on lung epithelial cells of the replicating group was significantly lower than that of the recovered group (Fig. 1*B*). Immunohistochemical assays further confirmed that the lungs of the replicating group were densely distributed with SARS-CoV-2 nucleocapsid (N) protein–expressing cells, in accordance with our nucleic acid test result, suggesting the active replication of SARS-CoV-2 as well as the significant lower expression of MHC-Ι (Fig. 1*C* and *SI Appendix*, Fig. S1*B*). Taken together, we found that SARS-CoV-2 infection led to MHC-Ι down-regulation both in vitro and in vivo.

Next, we identified the viral protein(s) of SARS-CoV-2 that may affect MHC-Ι expression. The genome of SARS-CoV-2 comprises ∼30,000 nucleotides, sharing 79% sequence identity with SARS-CoV. Similar to SARS-CoV, SARS-CoV-2 has four structural proteins: Spike (S), Envelope (E), Membrane (M), and Nucleocapsid (N) (22, 23). SARS-CoV-2 also harbors some accessory ORF proteins at its 3′ portion (Fig. 1*D*). As the function of almost all structural and nonstructural viral proteins of SARS-CoV has been identified, we reasoned that the possible HIV-1 Nef- or Vpu-like function, if it exists, would likely fall into the membrane-bound structural proteins or these 3′ accessory ORFs. First, we examined the structural proteins and unidentified ORFs of SARS-CoV-2 for possible anti-immune function. Among them, we found that ORF8 overexpression in the HEK293T cells significantly down-regulated MHC-Ι (HLA-A2) expression (Fig. 1*E*). The protein sequence of ORF8 of SARS-CoV-2 exhibited the least homology with that of SARS-CoV (Fig. 1*D*) (22–24). The sequence homology between SARS-CoV-2 and early-phase SARS-CoV (SARS-CoV_GZ02) in 2003, both of which contain a full-length ORF8, was ∼26% (*SI Appendix*, Fig. S1*C*). However, all SARS-CoV strains identified from the mid- and late-phase patients in 2003, such as SARS-CoV_BJ01, harbored a 29-nucleotide deletion that resulted in the splitting of ORF8 into ORF8a and ORF8b (*SI Appendix*, Fig. S1*C*). The SARS-CoV-2 ORF8 protein was more distant from ORF8a (at 10% sequence identity) and ORF8b (at 16% sequence identity) of SARS-CoV (SARS-CoV_BJ01) (*SI Appendix*, Fig. S1*C*). Neither ORF8a and ORF8b of SARS-CoV_BJ01 or intact ORF8 of SARS-CoV_GZ02 exerted an effect on MHC-Ι down-regulation (Fig. 1*F*). L84S mutation in the SARS-CoV-2 ORF8 protein was significant for genotyping and phylogenetic analysis (25, 26). Nonetheless, both L and S subtypes of SARS-CoV-2 ORF8 exerted similar effects in down-regulating MHC-Ι (Fig. 1*G*).

**Fig. 2.**
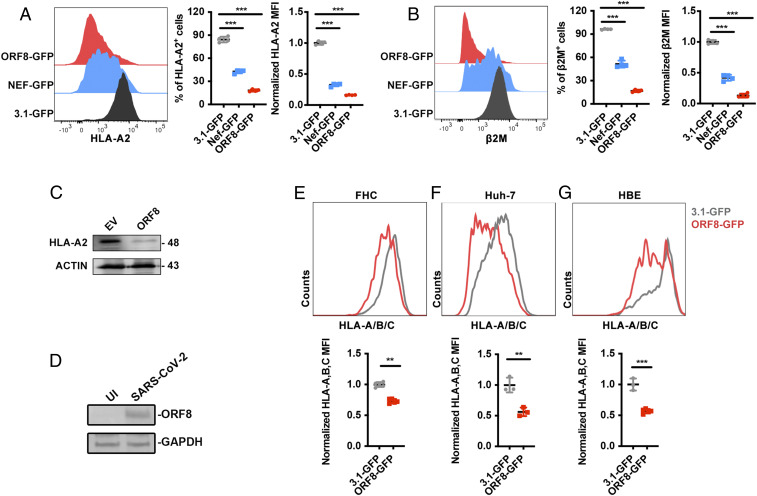
ORF8-induced MHC-Ι down-regulation. (*A* and *B*) 3.1-GFP (negative control), ORF8-GFP, or HIV-Nef-GFP (positive control)–expressing plasmid was transfected into HEK293T cells, respectively. Cells were collected at 48 h after transfection for flow cytometry analysis. Frequency and mean fluorescence intensity (MFI) of HLA-A2^+^ and β_2_-microglobulin (β_2_M)^+^ cells (gated on GFP^+^ cells) were shown (*n* = 5). MFI was normalized to GFP group. (*C*) Western blot analysis for *A* (*n* = 3). (*D*) HEK293T/Hace2 cells were infected with SARS-CoV-2 (hCoV-19/CHN/SYSU-IHV/2020) (MOI = 0.1). At 48 h after infection, cells were collected for Western blot (*n* = 3). (*E*–*G*) GFP- (negative control) or ORF8-GFP–expressing plasmids were transfected into FHC, HBE, or Huh7 cells, respectively. At 48 h after transfection, cells were harvested for flow cytometry analysis (gated on GFP^+^ cells) (*n* = 3) and normalized to GFP group. The data were shown as mean ± SD (error bars). Student’s *t* test and one-way ANOVA was used. *P* < 0.05 indicates statistically significant difference; **P < 0.01; ***P < 0.001.

To further confirm the effect of ORF8 in down-regulating MHC-Ι expression, an ORF8-expressing plasmid with separate green fluorescent protein (GFP) expression (ORF8-GFP) and 3.1-GFP were constructed and transfected into HEK293T cells. A HIV-1-Nef–expressing plasmid with separate GFP (Nef-GFP), which was previously constructed by us, served as a positive control (27). The cell surface expression of MHC-Ι heavy chain and the second polypeptide component of MHC-Ι complex β_2_-microglobulin (β_2_M) was determined using flow cytometry (*SI Appendix*, Fig. S1*D*). Compared with the 3.1-GFP, we found that the frequency and mean fluorescence intensity of MHC-Ι and β_2_M were significantly down-regulated by ORF8 overexpression (Fig. 2 *A* and *B*). The total protein expression of MHC-Ι was also significantly down-regulated (Fig. 2*C*). This effect was dose and time dependent (*SI Appendix*, Fig. S1 *E* and *F*). The protein expression of ORF8 in authentic SARS-CoV-2 strain–infected HEK293T/Hace2 cells was confirmed by Western blotting, which is consistent with recent proteomics data (28) (Fig. 2*D*). Furthermore, the expression of MHC-I molecules in various cell lines (i.e., human fetal colon cell line (FHC), human bronchial epithelial cell line (HBE), and human liver cell line (Huh7) were significantly down-regulated by ORF8 compared to 3.1-GFP (Fig. 2 *E*–*G*).

### ORF8 Knockdown Restores MHC-Ι Expression upon SARS-CoV-2 Infection.

**Fig. 3.**
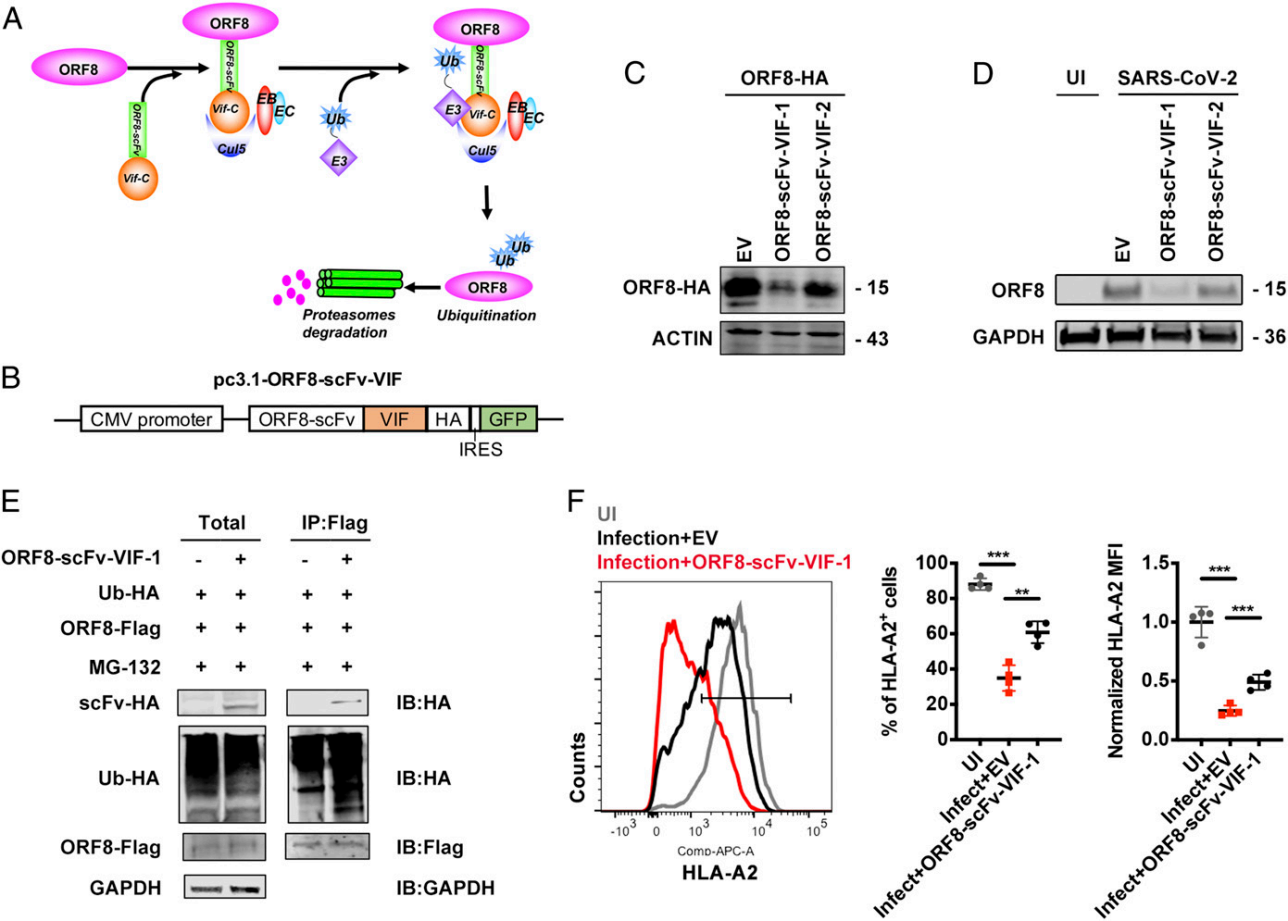
ORF8 knockdown restores MHC-Ι expression. (*A*) Schematic of the mechanism for ORF8-scFv-VIF to induce ORF8 degradation. (*B*) Schematic for construction of ORF8-scFv-VIF plasmid. (*C*) Cells were transfected with empty vector (EV), pORF8-scFv-VIF-1, or pORF8-scFv-VIF-2 in combination with ORF8-HA–expressing plasmid. At 48 h after transfection, cells were collected for Western blot (*n* = 3). (*D*) HEK293T cells transfected with or without ORF8-scFv-VIF were infected with SARS-CoV-2 (hCoV-19/CHN/SYSU-IHV/2020) (MOI = 0.1). At 48 h after infection, cells were collected for Western blot (*n* = 3). (*E*) ORF8 was co-IP with the overexpressed ORF8-scFv-VIF-1. Cells were transfected with ORF8-Scfv-1– or GFP (EV)-expressing plasmids together with ORF8-Flag– and Ubiquitin-HA–expressing plasmids and treated with MG132 (10 μM) for 12 h before harvest. Cells were collected at 48 h after transfection for co-IP with the anti–Flag-tag beads and detected with the indicated antibodies (*n* = 5). (*F*) The ACE2-expressing HEK293T cells (HEK293T/Hace2) transfected with EV or ORF8-scFv-VIF-1 were infected with SARS-CoV-2 (hCoV-19/CHN/SYSU-IHV/2020) (MOI = 1). At 48 h after infection, cells were collected for flow cytometry analysis (*n* = 3). Mean fluorescence intensity was normalized to uninfected group. The data were shown as mean ± SD (error bars). Student’s *t* test and one-way ANOVA was used. *P* < 0.05 indicates statistically significant difference; **P < 0.01; ***P < 0.001.

To knock down ORF8 expression in SARS-CoV-2–infected cells, we attempted to use ORF8-specific small interfering RNAs (siRNAs) to decrease ORF8 subgenomic RNA. However, the efficiency was not satisfactory. Therefore, we used a knockdown system at the protein level, which was developed by us and others, to induce the specific degradation of ORF8 protein using an engineered E3 ubiquitin-protein ligase (Fig. 3*A*) (29, 30). An anti-ORF8 scFv (ORF8-scFv-1) was enriched through four rounds of phage-display panning against ORF8 protein. ORF8-scFv-1 was then fused to the C terminus of HIV-1 Vif, which can interact with Elongin B, Elongin C, and Cul5, leading to ubiquitin-proteasome system (UPS)-mediated degradation of its natural target APOBEC3G and the artificial target protein Kras (29, 31) (*SI Appendix*, Table S1). After the plasmids expressing ORF8-scFv-VIF-1 and ORF8-scFv-VIF-2 were constructed (Fig. 3*B*), they were transfected into ORF8-overexpressing HEK293T cells or HEK293T cells that were subsequently infected by SARS-CoV-2 viruses. We found that ORF8-scFv-VIF-1 significantly knocked down ORF8 protein expression in both types of cells (Fig. 3 *C* and *D*). Furthermore, we confirmed that ORF8-scFv-VIF-1 can directly bind to ORF8 and induce ubiquitination in the presence of MG132, suggesting that ORF8-scFv-VIF-1 mediated ORF8 degradation through the UPS pathway (Fig. 3*E*). Notably, upon the knockdown of ORF8 by ORF8-scFv-VIF-1, the down-regulation of surface MHC-Ι expression induced by authentic SARS-CoV-2 virus was rescued (Fig. 3*F*). Taken together, we showed that SARS-CoV-2 infection down-regulates MHC-Ι potently through ORF8.

### MHC-Ι Is Selectively Targeted for Lysosomal Degradation by ORF8.

**Fig. 4.**
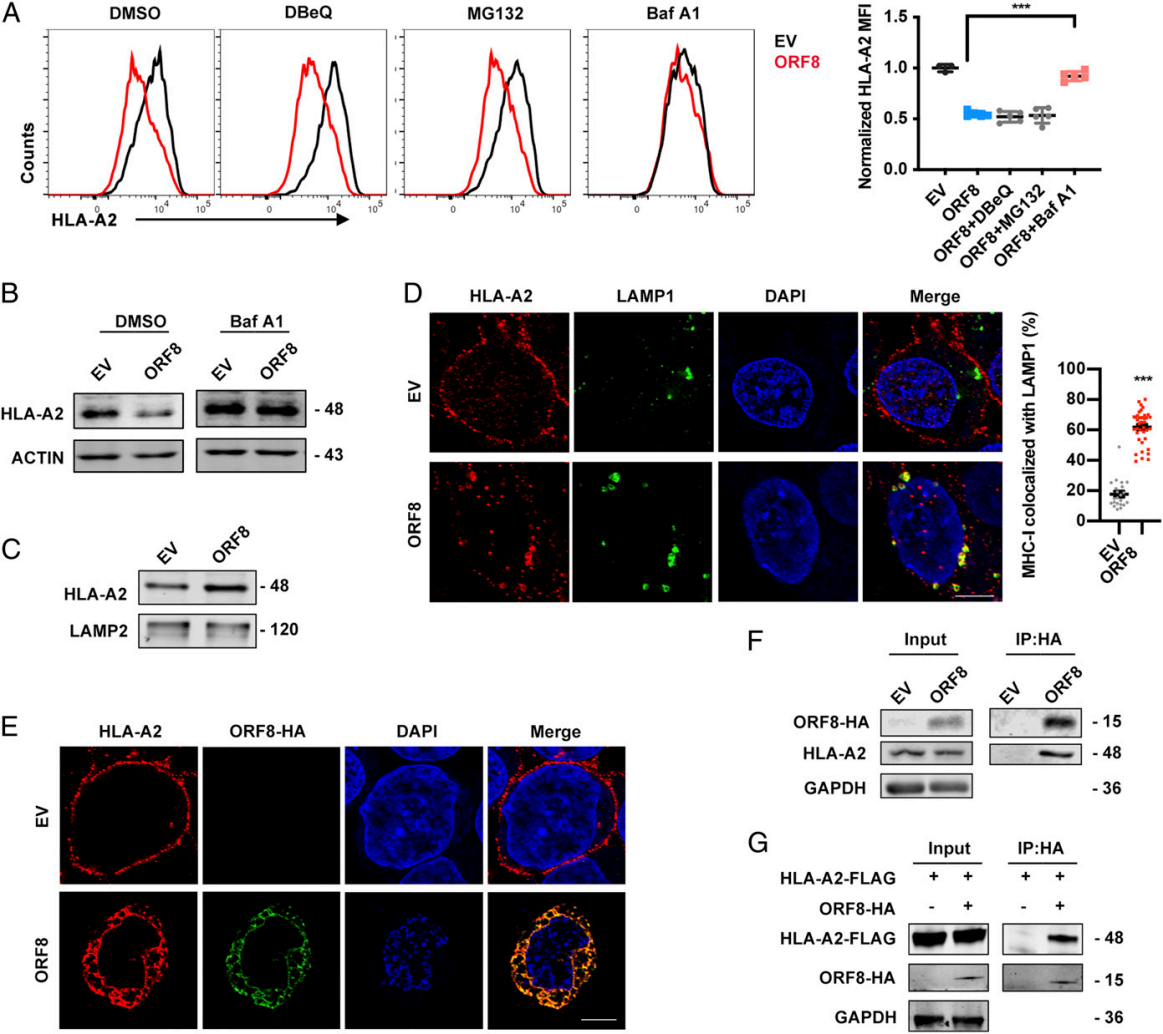
MHC-Ι is targeted for lysosomal degradation by ORF8. (*A* and *B*) GFP- (EV) or ORF8-GFP–expressing plasmid was transfected into HEK293T cells. Before harvest, cells were treated with DMSO and DBeQ (15 μM) for 4 h, MG132 (10 μM) for 4 h, and bafilomycin A1 (Baf A1, autophagy inhibitor, 100 nM) for 16 h. Cells were collected at 36 h after transfection and HLA-A2 mean fluorescence intensity was analyzed by flow cytometry (gated on GFP^+^ cells) and normalized to GFP (EV) group, and the total HLA-A2 protein expression was analyzed by Western blotting (*n* = 5). (*C*) Cells transfected with empty vector (EV)- or ORF8-HA–expressing plasmid were treated with Baf A1 (100 nM) for 16 h before harvest for crude lysosomal fraction. Accumulation of HLA-A2 in lysosomes was analyzed by Western blotting (*n* = 5). (*D*) Localization of HLA-A2 (red) relative to LAMP1-positive (green) lysosomes (Scale bars, 5 μm). Cells were transfected with EV- or ORF8-HA–expressing plasmid. At 24 h after transfection, colocalization was visualized by confocal microscopy (*n* = 20 to 40 fields). (*E*) Localization of HLA-A2 (red) relative to SARS-CoV-2 ORF8-HA (green) (Scale bars, 5 μm). Cells were transfected with EV- or ORF8-HA–expressing plasmid. At 16 h after transfection, colocalization was visualized by confocal microscopy (*n* = 14 to 20 fields). (*F*) ORF8 was co-IP with HLA-A2. EV- or ORF8-HA–expressing plasmid was transfected into HEK293T cells, respectively. Cells were treated with Baf A1 (100 nM) for 16 h before being collected. The cells were collected at 48 h after transfection and treated with cross-linker DSP and co-IP with the anti–HA-tag beads (*n* = 5). (*G*) ORF8 was co-IP with the overexpressed HLA-A2. Cells were transfected with HLA-A2-FLAG–expressing plasmid together with ORF8-HA–expressing plasmid or vector and treated with Baf A1 (100 nM) for 16 h before harvest. Cells were collected at 48 h after transfection for co-IP with the anti–HA-tag beads (*n* = 5). The data were shown as mean ± SD (error bars). *t* test and one-way ANOVA was used. *P* < 0.05 indicates statistically significant difference; ***P < 0.001.

To elucidate the mechanism of ORF8-mediated MHC-Ι down-regulation, the cells were treated with different inhibitors that block membrane protein degradation via different pathways, including N2, N4-dibenzylquinazoline-2,4-diamine (DBeQ), which blocks endoplasmic reticulum (ER)-associated protein degradation (ERAD); MG132, which blocks UPS; and bafilomycin A1 (Baf-A1), which blocks lysosomal degradation. Among these inhibitors, the most significant counteraction of MHC-Ι protein expression reduction by ORF8 was mediated by Baf-A1, suggesting that lysosomal degradation is the major pathway for ORF8-mediated MHC-Ι down-regulation (Fig. 4 *A* and *B*). Indeed, we found that MHC-Ι was enriched in lysosomes in ORF8-expressing cells (Fig. 4*C*). Furthermore, at 24 h after ORF8 transfection, surface MHC-Ι expression was almost abrogated and redistributed into the cytoplasm, demonstrating a strong colocalization with LAMP1 (Fig. 4*D*). We further determined whether ORF8 and MHC-Ι could interact physically. At 16 h after ORF8 transfection, we found that ORF8 strikingly colocalized with MHC-Ι by performing confocal microscopy (Fig. 4*E*). Immunoprecipitation data further confirmed the binding of ORF8 with either endogenous or exogenous MHC-Ι (Fig. 4 *F* and *G*). Collectively, these data suggest that ORF8 directly binds to MHC-Ι molecules and targets them for lysosomal degradation.

### ORF8 Mediates MHC-Ι Degradation through Beclin 1–Mediated Autophagy Pathway.

**Fig. 5.**
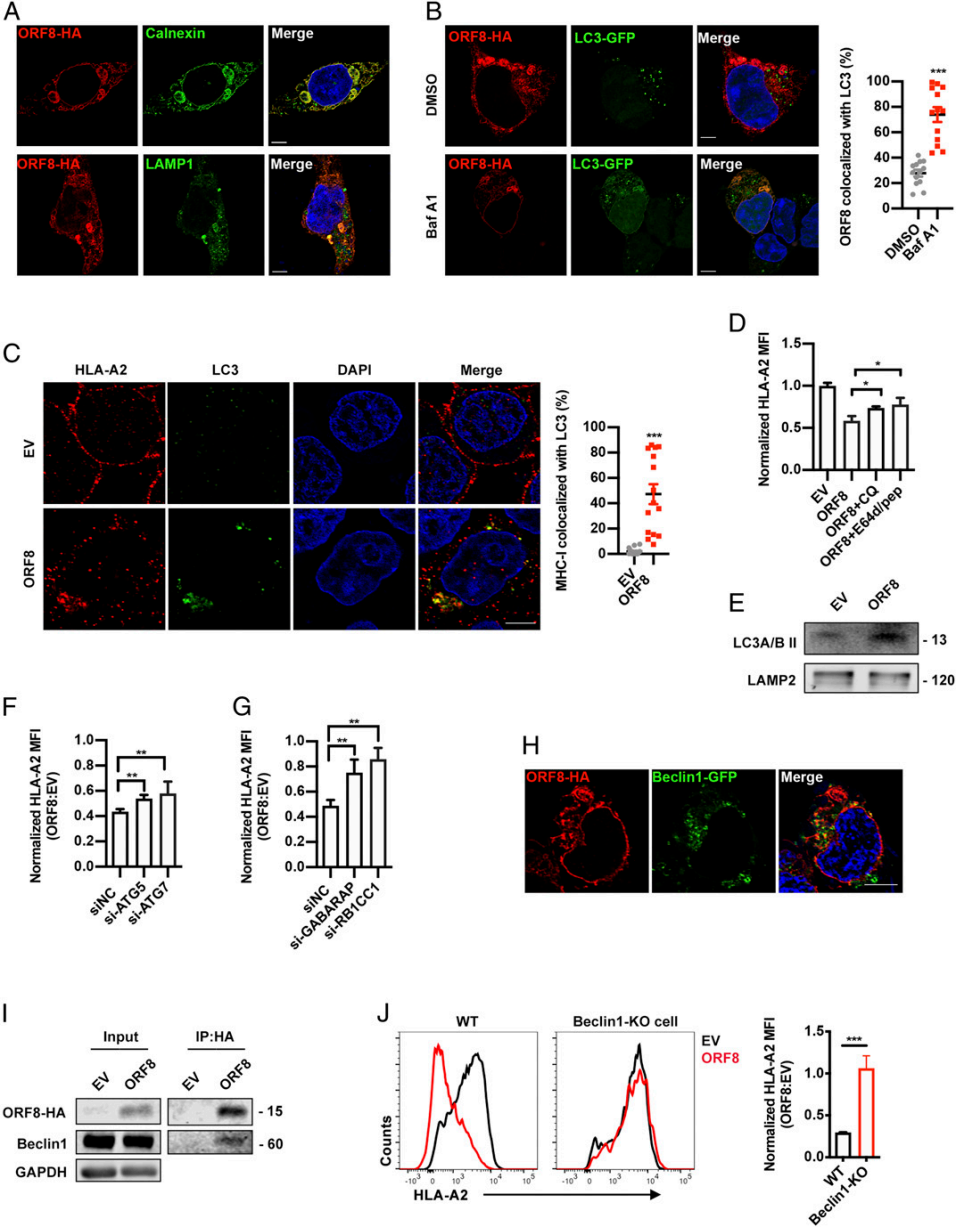
ORF8 mediates MHC-Ι degradation through autophagy pathway. (*A*) Localization of SARS-CoV-2 ORF8-HA (red) relative to CALNEXIN (green, *Top*) or LAMP1 (green, *Bottom*). ORF8-HA–expressing plasmid was transfected into HEK293T cells. At 24 h after transfection, colocalization was visualized by confocal microscopy (Scale bars, 5 μm). (*B*) Localization of SARS-CoV-2 ORF8 (red) relative to LC3-GFP (green). ORF8-HA– and LC3-GFP–expressing plasmids were cotransfected into HEK293T cells. At 24 h after transfection, colocalization was visualized by confocal microscopy (Scale bars, 5 μm) (*n* = 14 to 20 fields). (*C*) Localization of HLA-A2 (red) relative to LC3 (green). ORF8-HA–expressing plasmids were transfected into HEK293T cells. At 24 h after transfection, colocalization was visualized by confocal microscopy (Scale bars, 5 μm) (*n* = 14 to 20 fields). (*D*) GFP- (empty vector, EV) or ORF8-GFP–expressing plasmid was transfected into HEK293T cells. Before harvest, cells were then treated with chloroquine (CQ) (50 μM) and E64d (10 μg/mL) and pepstatin A (pep) (10 μg/mL) for 6 h. The HLA-A2 mean fluorescence intensity (MFI) (gated on GFP^+^ cells) was normalized to GFP group (*n* = 5). (*E*) EV- or ORF8-HA–expressing plasmid was transfected into HEK293T cells. Cells were treated with Baf A1 (100 nM) for 16 h before harvest for crude lysosomal fraction. Accumulation of LC3B in lysosomes was analyzed by Western blotting. (*F* and *G*) GFP (EV)- or ORF8-GFP–expressing plasmids and the indicated siRNAs were transfected into HEK293T cells. MFI of HLA-A2 (gated on GFP^+^ cells) was normalized to GFP group (*n* = 5). (*H*) Localization of SARS-CoV-2 ORF8-HA (red) relative to Beclin 1-GFP (green) (Scale bars, 5 μm). ORF8-HA– and Beclin 1-GFP–expressing plasmids were cotransfected into HEK293T cells. At 16 h after transfection, colocalization was visualized by confocal microscopy (*n* = 14 to 20 fields). (*I*) ORF8 was co-IP with Beclin 1. Empty vector (EV)-, or ORF8-HA–expressing plasmid was transfected into HEK293T cells, respectively. Cells were treated with Baf A1 (100 nM) for 16 h before collected. The cells were collected at 48 h after transfection and treated with cross-linker DSP and co-IP with the anti–HA-tag beads (*n* = 5). (*J*) GFP (EV)- or ORF8-GFP–expressing plasmids were transfected into HEK293T cells (WT), or Beclin 1 knockout HEK293T cells. Cells were collected at 48 h after transfection, and HLA-A2 MFI was analyzed by flow cytometry (gated on GFP^+^ cells) and normalized to GFP (EV) group (*n* = 5). The data were shown as mean ± SD (error bars). Student’s *t* test and one-way ANOVA was used. *P* < 0.05 indicates statistically significant difference; *P < 0.05; **P < 0.01; ***P < 0.001.

To determine how SARS-CoV-2 down-regulates MHC-Ι expression through ORF8, we performed mass spectrometry analysis to identify the proteins interacting with ORF8 protein. Consistent with a previous report (32), the top enrichments of SARS-CoV-2 ORF8–interacting proteins were observed in the ER, indicating that the host interactions of ORF8 may facilitate significant reconfiguration of ER trafficking during viral infection (*SI Appendix*, Fig. S2*A*). In addition, ORF8 showed strong colocalization with CALNEXIN^+^ ER and LAMP1^+^ lysosome rather than GM130^+^ Golgi or RAB5^+^ early endosome (Fig. 5*A* and *SI Appendix*, Fig. S2 *B* and *C*), suggesting that ORF8 most likely down-regulated MHC-Ι expression in the ER or lysosome rather than in the Golgi or plasma membrane. Moreover, the knockdown of vesicle-trafficking–related AP1, AP2, or AP3 proteins failed to counteract MHC-Ι down-regulation mediated by ORF8, excluding the possible involvement of vesicles in cargo transport from the trans-Golgi network, plasma membrane, or endosomal network (*SI Appendix*, Fig. S3 *A* and *B*) (33). Conversely, the knockdown of ERAD-related proteins (i.e., HDR1, SEL1L1, ERLIN2, CANX, OS9, or ERLEC1) failed to counteract ORF8-mediated MHC down-regulation (*SI Appendix*, Fig. S3 *C* and *D*) (34). MHC-Ι ubiquitination did not significantly change following ORF8 overexpression, excluding the possible involvement of ERAD pathway (*SI Appendix*, Fig. S3*E*). Thus, we presumed that ORF8 could mediate MHC-Ι trafficking from the ER to lysosomes for degradation. Trafficking from ER to lysosomes is most likely mediated by ER-phagy, which is a type of selective autophagy. Six autophagy cargo receptor proteins bind to and recruit substrates to autophagosomal membranes (35). To examine their possible involvement, these receptors (i.e., FAM134B, RTN3, ATL3, SEC62, CCPG1, and TEX264) were knocked down with siRNAs, respectively. However, we did not observe any effect on MHC-Ι expression in the presence of ORF8 (*SI Appendix*, Fig. S3 *F* and *G*).

Nevertheless, to determine the possible involvement of autophagy, we first examined the colocalization between ORF8 or MHC-Ι and autophagosomes within the cells. A substantial fraction of ORF8 colocalized with LC3B-labeled autophagosomes in ORF8-expressing cells (Fig. 5*B*). A substantial fraction of the MHC-Ι puncta also colocalized with LC3B-labeled autophagosomes (Fig. 5*C*). Furthermore, the specific autophagy inhibitors chloroquine and E64/pep restored MHC-Ι expression on cell surface and restored its total protein level (Fig. 5*D* and *SI Appendix*, Fig. S4*A*). LC3B was also highly enriched in lysosomes in ORF8-expressing cells (Fig. 5*E*). Specifically, we found that the knockdown of ATG5, ATG7, and the autophagy cargo proteins RB1CC1 (FIP200) or GABARAP restored MHC-Ι expression both on the cell surface and total protein level (Fig. 5 *F* and *G* and *SI Appendix*, Fig. S4 *B*–*D*).

Furthermore, we found that a substantial fraction of ORF8 colocalized with Beclin 1, an essential protein for autophagy initiation to mediate autophagosome formation (Fig. 5*H*) (36, 37). Immunoprecipitation data further confirmed that ORF8 directly interacted with Beclin 1 (Fig. 5*I*). In the Beclin 1–knockout HEK293T cell line, ORF8 failed to induce surface MHC-Ι degradation, while the HIV-Nef transfection still down-regulated surface MHC-Ι expression (Fig. 5*J* and *SI Appendix*, Fig. S4 *E* and *F*). However, the knockdown of NBR1, which is involved in MHC-Ι down-regulation in pancreatic cancer cells, did not exert any effect, excluding NBR1 as a receptor for ORF8-mediated MHC-Ι degradation (*SI Appendix*, Fig. S4 *G* and *H*) (38). The knockdown of several other possible receptors with siRNAs also did not exert any effect on ORF8-mediated MHC-Ι down-regulation (*SI Appendix*, Fig. S4 *G* and *H*) (39). Taken together, our results suggested that ORF8 could hijack the Beclin 1 autophagy initiation pathway and thus mediate MHC-Ι degradation through autophagy.

### ORF8 Enables SARS-CoV-2 to Be Less Sensitive to Antiviral CTLs.

**Fig. 6.**
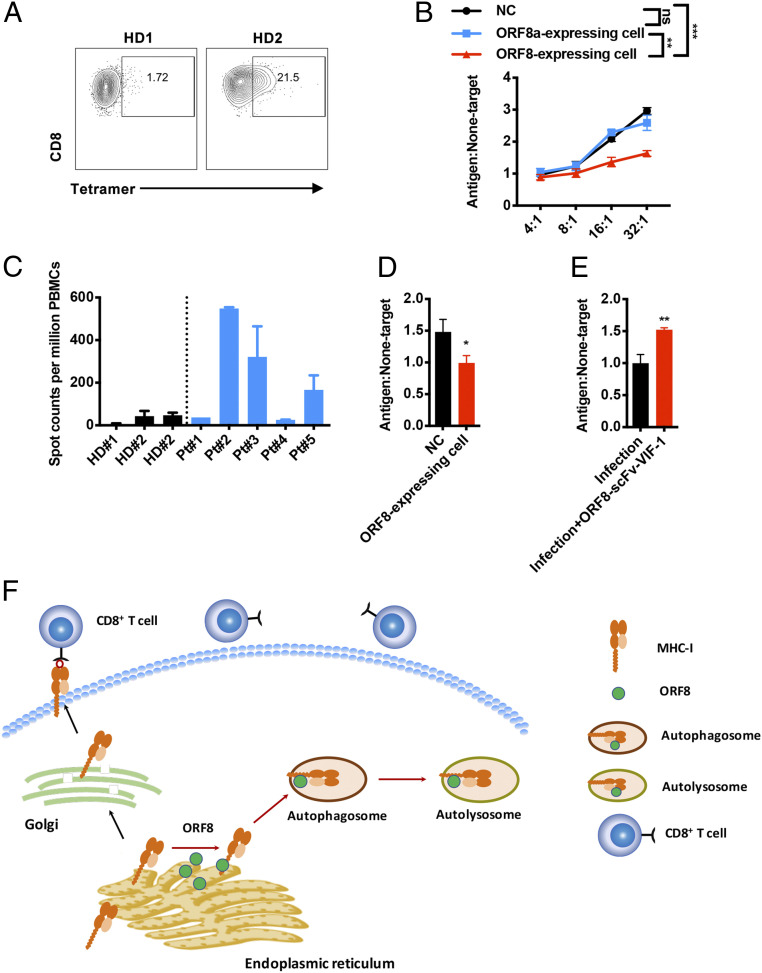
ORF8-mediated resistance of SARS-CoV-2 to antiviral CTLs. (*A*) Frequency of SSp-1–specific CD8^+^ T cells (gated on CD8^+^ cells) generated from HLA-A2^+^ healthy donors (HD). (*B*) Killing assay using SSp-1–specific CD8^+^ T cells generated form HD. CTLs were cocultured with SSp-1 peptide–loaded HEK293T cells (antigen), or with HIV-gag peptide (SL9)-loaded HEK293T cells (nontarget) overnight. The ratios of dead target versus nontarget cells (antigen: nontarget) were determined by flow cytometry. (*C*) IFN-γ ELISpot analysis of COVID-19 recover patients (Pt) to synthetic peptides, compared to HD. (*D*) Killing assay using CD8^+^ T cells from HLA-A2^+^ COVID-19–recovered Pt 3. Activated CTLs were cocultured with SARS-CoV-2 peptide –loaded HEK293T cells (antigen) or with HIV-gag peptide–loaded HEK293T cells (nontarget) at effector: target ratio 8:1. The ratios of dead target versus nontarget cells (antigen: nontarget) were determined by flow cytometry (*n* = 5). (*E*) Killing assay for authentic SARS-CoV-2–infected HEK293T/Hace2 cells using SSp-1–specific CD8^+^ T cells generated form HD. CTLs were cocultured with infected HEK293T/Hace2 cells (antigen) or uninfected cells (nontarget) at effector: target ratio 30:1. The ratios of dead target versus nontarget cells (antigen: nontarget) were determined by flow cytometry. (*F*) Schematics showing that ORF8 mediates MHC-Ι lysosome degradation through an autophagy-dependent pathway. In SARS-CoV-2–infected cells, ORF8 directly binds to the MHC-Ι molecule, facilitating its trafficking to autophagosome for lysosome degradation. The data were shown as mean ± SD (error bars). Student’s *t* test was used. *P* < 0.05 indicates statistically significance difference; *P < 0.05; **P < 0.01; ***P < 0.001.

It is known that CTLs are involved in immune-mediated protection against coronavirus infection (40). Down-regulation of MHC-Ι by ORF8 can result in impairment of CTL-mediated killing of SAR-CoV-2–infected cells. SSp-1 is predicted to be a potential SARS-CoV-2 epitope (41) that is well characterized for immune response (42). To investigate the immune evasion caused by ORF8-mediated MHC down-regulation, we generated SSp-1–specific CTLs by sensitization of HLA-A2^+^ healthy donor peripheral blood lymphocytes (PBLs) with autologous dendritic cells prepulsed with SSp-1 (Fig. 6*A*). SSP-1 pulsed control HEK293T cells or ORF8-expressing HEK293T cells were used as target cells. The result showed that SSp-1–specific CTLs eliminated SARS-CoV-2 ORF8–expressing target cells with lower efficiency, compared to wild-type or SARS-CoV ORF8a–expressing target cells (Fig. 6*B*). Furthermore, we isolated SARS-CoV-2–specific CD8^+^ T cells from five patients who recently recovered from the infection. Among these five patients, patient no. 2, 3, and 5 showed strong antigen-specific T cell response to SARS-CoV-2 S peptides (Fig. 6*C*). The CD8^+^ T cells of HLA-A2^+^ donor patient no. 3 was therefore used for CTL killing assay. ORF8-expressing target cells or wild-type controls were pulsed with the synthetic peptide mixture of SARS-CoV-2; they were then mixed with the CTLs. Compared with the control, the SARS-CoV-2–specific CTLs also eliminated ORF8-expressing target cells with lower efficiency, indicating that ORF8 protected the target cells from CTL-mediated lysis (Fig. 6*D*). Notably, ORF8 knockdown by ORF8-scFv-VIF-1 in SARS-CoV-2–infected cells restored sensitivity to CTL lysis (Fig. 6*E*). Collectively, these results strongly suggested that ORF8 mediates MHC-Ι down-regulation and protects the SARS-CoV-2–infected host cells from CTLs.

## Discussion

Viral infection elicits effective innate and adaptive immune responses to inhibit viral replication. The antiviral immunity against SARS-CoV-2 infection remains largely unknown. A proportion of recovered patients may still be virus carriers, and CD8^+^ lymphocyte dysfunction has been reported in these patients (7–9). These clinical characteristics of COVID-19 suggest that SARS-CoV-2 could lead to adaptive immune disorder while maintaining active viral replication. In this study, we demonstrated that ORF8 of SARS-CoV-2 mediates MHC-Ι down-regulation, which is not observed in other strains of SARS-CoV. As SARS-CoV-2 ORF8 is the only protein that shares approximate 20% homology with SARS-CoV, we suggest that ORF8 is a relatively new protein in SARS-CoV-2 compared with SARS-CoV. In this context, immune evasion mediated by ORF8 in SARS-CoV-2 can, at least in part, explain why the disease spectrum of COVID-19 is different from that of SARS.

In this study, we identified that ORF8 of SARS-CoV-2 could mediate the down-regulation of MHC-Ι both upon virus infection and plasmid transfection. ORF8 transfection could induce MHC-Ι down-regulation in a dose- and time-dependent way, and this effect is consistent in different experimental settings. Furthermore, by using HIV-1 Nef as the control, which is one of the well-known viral molecules to mediate immune evasion though MHC-I down-regulation, the down-regulation of MHC-I by SARS-CoV-2 ORF8 is similar to that of HIV-1 Nef in our experimental system. Moreover, the killing assay showed that the target cells significantly evaded from the recognition of CTLs in the presence of ORF8 at low concentration. Together, ORF8 is at least one explanation of the immune evasion caused by SARS-CoV-2 and is very worthy further study on clinical samples.

Antiviral T cell recognition of infected host cells is crucial for viral clearance. Virus-reactive T cells, including those that are ORF8-responsive, were detected in patients who had recovered from COVID-19 (43, 44). The MHC-Ι down-regulation by ORF8 could shield the virus from T cell antiviral immunity, as our in vitro CTL killing assay indicated. A dampened antiviral T cell response could lead to worsened symptoms and prolonged recovery. Consistent with our finding, a recent clinical study reported the discovery of an ORF8-defective SARS-CoV-2 strain (∆382) in Singapore. Although the ORF8-defective ∆382 SARS-CoV-2 strain exhibited similar virus replication kinetics, the patients infected with this strain developed attenuated clinical symptoms with none requiring oxygen supply, compared to the 28% of patients infected with the wild-type strain requiring oxygen supply. Moreover, higher IFN-γ, TNF-α, IL-2, and IL-5 were detected in patients infected with this viral strain versus wild-type virus, suggesting improved T cell recognition of infected cells in the absence of ORF8 (45). This report is real-world epidemiology data demonstrating how ORF8 could impact the severity of COVID-19 as well as T cell immune responses.

Although other viruses have also developed the ability to evade immune surveillance by impairing antigen presentation, their underlying mechanisms differ from each other. HIV-1 Nef mainly facilitates the interaction between AP-1 and MHC and prevents the movement of MHC-Ι molecule to the plasma membrane. Instead, it reroutes MHC-Ι from the trans-Golgi network to late endosomes/lysosomes for degradation (33, 46). K3 and K5 proteins of KSHV induce the ubiquitination of MHC-Ι on the plasma membrane and facilitate its endocytosis (14, 47). Moreover, E3/E19 protein encoded by adenovirus disrupts the association between the ER protein TAP and MHC-Ι and retains MHC-Ι molecule in the ER, thereby impairing the peptide–MHC-Ι assembly and presentation (48, 49). In line with previous reports, MHC-Ι was pulled down using SARS-CoV-2 ORF8 as bait, along with other host proteins in ER quality control and glycosaminoglycan synthesis (28). In the present study, we found that the ER-resident protein ORF8 induces MHC-Ι degradation. After excluding the possible involvement of ERAD and other abnormal trafficking, it can be assumed that autophagy is involved in this process.

Furthermore, we found that ORF8 and MHC-Ι are colocalized with LC3-labeled autophagosome. The inhibition of autophagy pathway by specific autophagy inhibitors or by specific knockdown with siRNAs targeting ATG5, ATG7, RB1CC1, or GABARAP significantly restored the surface MHC-Ι expression. These lines of evidence clearly indicate the involvement of autophagy. Although we did not find any evidence showing the involvement of the six identified ER-phagy receptors, we found that ORF8 interacted and colocalized with Beclin 1. Beclin 1 knockout restored MHC-Ι expression on the cell surface. It is well known that Beclin 1 interacts with many regulatory proteins and acts as a scaffold to form multiprotein complex. In addition, Beclin 1 is a key player during autophagy initiation and nucleation (36). Although many proteins (i.e., HIV-1 Nef or the influenza virus matrix protein 2) interact with Beclin 1, leading to the blockade of autophagosome initiation, some Beclin 1–interacting proteins (i.e., Ambra1, Atg14L (Barkor), UVRAG, HMGB1, and Rubicon) induce the activation or maturation of autophagosomes (36, 50, 51). Notably, ORF8 of SARS-CoV-2 is a unique example of pathogen-derived Beclin 1–binding protein that drives autophagy activation or maturation and further induces degradation of MHC-Ι of the host cells. Based on our findings, we propose that MHC-Ι on the ER is captured by ORF8 and subsequently connected to Beclin 1 instead of the regular routing through Golgi to plasma membrane, triggering the generation of autophagosomes for degradation (Fig. 6*F*).

Utilizing the SARS-CoV-2 antigen–specific CTLs as well as the CTLs from a COVID-19–convalescent patient for in vitro killing assay, we found that CTLs failed to effectively identify ORF8-expressing target cells or the SARS-CoV-2–infected target cells. Unlike HIV-1 Nef–mediated MHC-Ι degradation, which is one of the most well-established and thoroughly studied models for virus to mediate immune evasion, little is known about how SARS-CoV2 mediated immune evasion. Therefore, we used a surrogate in vitro model to investigate how ORF8 mediated evasion from T cell surveillance. In this model, HEK293T cells were either transiently transfected with ORF8 in coherence with our molecular biological models or infected by SARS-CoV2 followed by ORF8 protein–specific degradation via ORF8-ScFv-VIF-1. To eliminate the impact of transient transfection on cell viability, we adapted a target-to-nontarget ratio method to measure the CTL killing effect, an established methodology also applied in HIV and Middle East respiratory syndrome studies (52). We observed target cell killing by antigen-specific CTLs in a dose-dependent manner. However, ORF8-expressing target cells were less sensitive to CTL killing in both ORF8 transiently transfected and SARS-CoV2–infected models. Our model validated the ORF8-mediated evasion of antigen-specific CTL killing by MHC-Ι degradation. Although the clinical investigation regarding ORF8-defective SARS-CoV-2 strain in Singapore is highly compatible with our hypothesis, the ORF8-defective SARS-CoV-2 strain is a subject of ongoing and future studies, including in vivo studies (45).

SARS-CoV-2 utilizes its ORF8 protein as a unique mechanism to alter the expression of but not limited to surface MHC-Ι to evade immune surveillance. It subtly employs the autophagy pathway, which usually functions as an antiviral strategy, to reach its purpose. Although the detailed molecular mechanisms remain to be elucidated, our findings provide an important aspect for understanding how ORF8 impairs the antigen presentation system and assists in SARS-CoV-2 immune evasion. The current anti-SARS-CoV-2 drugs mainly target the enzymes or structural proteins essential to viral replication. Our study may provide evidence to promote the development of compounds specifically targeting the impairment of MHC-Ι antigen presentation by ORF8, thereby enhancing immune surveillance against SARS-CoV-2 infection.

## Methods

### Ethics Statement and Patient Cohort.

This research was approved by the Ethics Review Board of The Fifth Affiliated Hospital of Sun Yat-sen University and Sun Yat-Sen University. The five patients who recently recovered from SARS-CoV-2 infection were recruited for this study from The Fifth Affiliated Hospital of Sun Yat-sen University. The given written informed consent with approval of the Ethics Committees was accomplished before the study. Unidentified human peripheral blood mononuclear cells (PBMCs) of healthy blood donors provided by the Guangzhou Blood Center. We did not have any interaction with these human subjects or protected information, and therefore no informed consent was required. All animal experiments were carried out in strict accordance with the guidelines and regulations of Laboratory Monitoring Committee of Guangdong Province of China and were approved by Ethics Committee of Zhongshan School of Medicine of Sun Yat-sen University on Laboratory Animal Care. Viral infections were performed in a biosafety level 3 (BSL3) facility in accordance with recommendations for the care and use of laboratory animals.

### Cell Lines.

HEK293T, Huh7, and Vero E6 cell lines was obtained from the American Type Culture Collection. FHC and HBE cell lines are kindly gifted from Wen Liu, Sun Yat-sen University, Guangzhou, Guangdong, China (53). Theses cell lines conducted authentication through short tandem repeat profiling, karyotyping and cytochrome *c* oxidase I testing. Test for bacterial and fungal contamination was carried out by using current United States Pharmacopeia methods for viral testing adhering to the United States Code of Federal Regulation (9 CFR 113.53) guidelines, while mycoplasma testing was carried out by direct culture and Hoechst DNA staining and Limulus amoebocyte lysate assay to measure endotoxin values. Cells were maintained in a humidified incubator at 37 °C with 5% CO_2_, grown in Dulbecco’s modified Eagle’s medium (DMEM) (Gibco) supplemented with 10% fetal bovine serum (FBS) (Gibco), 100 units/mL penicillin (Gibco), and 100 μg/mL streptomycin (Gibco).

### Sequence Data Collection and Alignment.

The sequences were collected from GenBank database (https://www.ncbi.nlm.nih.gov/nuccore/), including one from SARS-CoV-2_WHU01 (accession number MN988668), one from SARS-CoV-2_HKU-SZ (MN938384), one from SARS-CoV_BJ01 (AY278488), and one from SARS-CoV_GZ02 (AY390556). The sequence alignment of complete genome sequences was performed using MAFFT software with default parameters (54). The protein alignments were created by Clustal Omega software using default parameters conducted in MEGA X (55). The pairwise sequence identities were calculated using BioEdit software. The similarity analysis based on the genome sequence was performed using SimPlot software (56). The annotation of the genome of SARS-COV-2 was updated with the National Center for Biotechnology Information reference genome sequence (accession number NC_045512).

### Key Resource.

All the antibodies, chemicals, peptides, and recombinant proteins used in this paper were listed in *SI Appendix*, *Supplemental Materials*.

### SARS-CoV-2 Infection.

For HEK293T cells infection, HEK293T cells (1.6 × 10^5^ cells/mL) were transfected with pcCMV-ACE2-Flag with and without pORF8-scFv-VIF-1. After 24 h, cells were washed with phosphate-buffered saline (PBS) and infected with authentic SARS-CoV-2 for 1 h at 37 °C. Then, cells were washed with PBS and replaced with DMEM (2% FBS). At 48 h after infection, cells were harvested for Western blot or testing HLA-A2 expression with flow cytometry.

For hACE2 mice infection, Transgenic hACE2 mice (C57BL/6) were purchased from GemPharmatech Co,Ltd. Littermates of the same sex were randomly assigned to uninfection or infection groups. The infection was performed as previously described (16). Mice were anesthetized with isoflurane and inoculated intranasally with 4 × 10^3^ or 4 × 10^4^ PFU of SARS-CoV-2 viruses. The lungs were collected at day 6 after infection. The virus stocks were obtained from the supernatant of Vero E6 after inoculation for 48 h, and the titers were determined by plaque assay targeting N protein. Body weight and survival rates of each mice were measured daily.

### Histopathology and Immunohistochemistry.

SARS-CoV-2–challenged hACE2 mice were euthanized in BSL3 facility. Lung was collected and fixed in 4% paraformaldehyde buffer for 48 h, followed by embedding with paraffin, then stained with hematoxylin and eosin. For immunohistochemistry, lung sections of each mouse were deparaffinized and rehydrated with xylene and gradient alcohol. The antigen was microwave retrieved by citric acid buffer (pH = 6.0) and then quenched for endogenous peroxidases with 3% H_2_O_2_ for 10 min. Bovine serum albumin (BSA) was used to block nonspecific binding sites at room temperature for 30 min. The sections were incubated with rabbit anti–SARS-CoV-2 N (Sinobiological) and H2Kb (Biolegend) at 1:200 dilution for overnight at 4 °C. Subsequently, the sections were incubated with goat anti-rabbit IgG secondary antibody for 2 h at room temperature and stained by 3,3′-diaminobenzidine. Finally, the sections were dyed with hematoxylin, dehydrated with gradient concentrations of ethanol, cleared with xylene, and covered with neutral balsam for microscopic examination. Images were captured with Olympus BX63 microscope. Immunoreactivity was evaluated semiquantitatively based on staining intensity and proportion. The proportion of staining was scored from 0 to 3 as follows: 3, >50% of cells positive; 2, 10 to 49%; 1, <10%. Intensity of staining was scored from 0 to 3 (0, absent; 1, weak; 2, moderate; 3, intense). The immunoreactive score for each sample was determined by multiplying the intensity and the proportion of stained cells. Analysis was undertaken blindly without knowledge of treatment variables.

### Flow Cytometry.

For analysis of surface markers, cells were stained in PBS containing 0.5% (wt/vol) BSA with indicated antibodies. Surface proteins were stained for 30 min with the relevant fluorochrome-conjugated monoclonal antibodies and the LIVE/DEAD Fixable Viability Dyes (Thermo Scientific) in PBS containing 0.5% BSA on ice. The following antibodies were used: anti-HLA-A2 (BB7.2), anti-mouse H-2Kb/H-2Db (28-8-6), anti-mouse EpCAM (G8.8), anti-HLA-A,B,C (W6/32), anti-human β2-microglobulin (2M2), and anti-CD8a (53-6.7). Flow cytometry data were acquired on LSR Fortessa (Becton Dickinson).

### Plasmids.

The DNA sequences of SARS-CoV-2 structural proteins and ORFs tagged with HA were chemically synthesized in GENEWIZ and inserted into pcDNA3.1 vector. The ORF8 S mutant–expressing plasmid was constructed via a PCR-based mutagenesis method from pcDNA3.1-ORF8-HA by introducing a point mutation (L to S) at the 84 amino acid. The GFP coding sequence was at the 3′ terminus and constructed into the pcDNA3.1 vector (57). The IRES-GFP sequence was inserted into 3′ ORF8-HA and named ORF8-GFP. The HIV-Nef-GFP and ubiquitin-HA–expressing plasmid was used as previously described by us (27). pCMV LC3–GFP was a gift from Ersheng Kuang, Sun Yat-sen University, Guangzhou, Guangdong, China. The pCMV3-HLA-A-Flag, pCMV-ACE2-Flag, and pCMV3-Rab5-Myc were purchased from Sino Biological. The DNA sequence of ORF8-scFv-1 and ORF8-scFv-2 were chemically synthesized in Gene Create. The sequence of VIF C terminus tagged with HA was amplified with PCR and connected to ORF8-scFv-1 or ORF8-scFv-2 for fusion expression, then inserted into pcDNA3.1-IRES-GFP vector named ORF8-scFv-VIF-1 or ORF8-scFv-VIF-2 (29). All constructs were verified by DNA sequencing.

### Phage Display Screening of Anti–ORF8 scFv.

The human-sourced scFv phage display library was constructed in pCANTAB-5E vector by insertion of randomized codons in CDR1, CDR2, and CDR3 regions of heavy chain and light chain. The recombinant His-tagged ORF8 of SARS-CoV-2 was expressed in *Escherichia coli* by pET28a expression system and purified with nickel beads. The phage panning was performed according to previous studies (58). Briefly, the scFv phages were incubated with the ORF8-His–conjugated nickel beads; after three times washing by PBS, the binding phages were eluted and amplified. This panning procedure was repeated four times. Through these procedures, 16 phagy clones were isolated, and their binding affinity was validated by phage enzyme-linked immunosorbent assay. As a result, two phage clones with scFv sequences named ORF8-SCFV-1 and ORF8-SCFV-2 were obtained, which can specifically bind ORF8 protein with a high binding activity (59).

### siRNA Transfection.

siRNAs targeting indicated human genes and negative control siRNA were purchased from RiboBio. Three siRNAs were synthesized for each gene. The siRNAs targeting each gene were transfected as a mixture and have been validated by the company to ensure that at least one siRNA was able to knock down target gene messenger RNA up to 70%. At 12 h after cell seeding, cells were transfected with specific siRNAs targeting each gene using Lipofectamine RNAiMAX (ThermoFisher) according to the manufacturer’s instruction. At 12 h after siRNA transfection, ORF8-GFP plasmid was transfected. Each gene was set three biological replicates. At 48 h after siRNA transfection, cells were collected for Western blot and flow cytometry.

### Immunofluorescence Assay.

Immunofluorescence (IF) assay was performed as previously described (60). HEK293T cells were seeded on in μ-slide chambered coverslips (Ibidi; 80826) and transfected as indicated. Cells were collected at indicated time and washed with PBS and fixed with 4% poly-formaldehyde in room temperature for 10 min, then permeabilized with 0.1% Saponin in PBS for 15 min and blocked with 5% BSA PBS for 30 min. Cells were incubated with primary antibodies at room temperature for 1 h. After washing with 0.1% Tween-20 PBS three times, cells were stained with secondary antibodies for 1 h and 4′,6-Diamidino-2-phenylindole dihydrochloride (DAPI) for 5 min. Samples were scanned with Zeiss LSM880 confocal microscopy and analyzed with Imaris. Primary antibodies used in IF assay include anti-GM130 (CST), anti-Calnexin (Proteintech), anti-Rab5 (CST), anti-HA (MBL), anti-Lamp1 (CST), and anti-HLA-A2 (MBL). Images were obtained with LSM880 confocal microscopy (Zeiss). Image analysis and quantification were performed with Imaris 8.4 software (Bitplane).

### Lysosome Isolation.

For lysosome isolation experiments, HEK293T cells were transfected with indicated plasmids. The cells were treated with 10 μg/mL E64d and 10 μg/mL pepstatin A for 6 h. At 48 h after transfection, cells were collected for lysosome isolation. The preparation of crude lysosomal fraction was performed by following the manufacturer’s instructions (Sigma-Aldrich, LYSISO1). In brief, the 2.7 PCV of 1× extraction buffer was added into the cells. The lysis samples were vortexed to achieve an even suspension and then broken in a 7-mL Dounce homogenizer using Pestle B. Trypan blue solution staining was used to ascertain the degree of breakage. The samples were centrifuged at 1,000 × *g* for 10 min, and the supernatant was transferred to a new centrifuge tube. The samples were centrifuged again at 20,000 × *g* for 20 min in microcentrifuge tubes, and the supernatant liquid was removed.

### In-Cell Cross-Linking.

In-cell cross-linking was performed using dithiobis [succinimidyl propionate (DSP)] (Thermo Scientific) as previously described (61). DSP were freshly prepared as a 25-mM solution in dimethyl sulfoxide (DMSO) and diluted to a working concentration of 0.5 mM in PBS. Cells were washed twice with PBS and then incubated with the cross-linker solution for 30 min at room temperature. Then, cells were incubated at room temperature for 15 min with quenching solution (1M Tris-Cl, pH 7.5). Quenching solution was then removed, and cells were washed twice with PBS and cell lysates were prepared for coimmunoprecipitation assay.

### Coimmunoprecipitation.

Coimmunoprecipitation (co-IP) assay was performed as our previously described (57). In brief, HEK293T cells were lysed with Nonidet P-40 lysis buffer (10 mM Tris ⋅ HCl, pH = 7.4, 150 mM NaCl, 0.5% Nonidet P-40, 1% Triton X-100, 10% glycerol, 2 mM ethylenediaminetetraacetic acid, 1 mM Na3VO4, 1% protease inhibitor mixture (Sigma-Aldrich), and phosphatase inhibitor mixture (TargetMol) for 30 min on ice with briefly vortex every 10 min. During this period, anti-HA-tag beads were washed three times with ice-cold STN buffer (10 mM Tri-HCl buffered at pH 7.4, 150 mM NaCl, 0.5% Nonidet P-40, 0.5% Triton X-100). The lysates were collected and incubated with the prepared anti-HA-tag beads for 4 h or overnight at 4 °C with rotating. Then, the immunoprecipitates were washed 4 times with ice-cold STN buffer, eluted by boiling SDS loading buffer, and separated by SDS-PAGE for Western blotting or mass spectrometry analysis.

### Mass Spectrometry Analysis.

HEK293T cells were seeded on 10 cm dish and transfected with 12 μg of ORF8-HA, At 48 h after transfection, cells were collected and lysed for co-IP assay, and the elution was boiled at 100 °C with loading buffer supplemented with DTT and separated through 10% SDS-PAGE. The proteins were then visualized with ProteoSilver Plus Silver Stain Kit (Sigma-Aldrich) according to the manufacturer’s instructions. The whole lane was cut into 10 slices and prepared for liquid chromatography–tandem mass spectrometry analysis as previously described (60). Functional pathways representative of each gene signature was analyzed for enrichment in gene categories from the gene ontology biological processes database (Gene Ontology Consortium) using DAVID Bioinformatics Resources, observing correlation between two replicate experiments.

### Generation of CTLs in Healthy Donors.

The generation of CTLs was performed as previous reported (16). Briefly, the PBMCs derived from HLA-A2^+^ healthy donors were isolated from peripheral blood by Ficoll-Hypaque gradient separation. PBMCs were resuspended in Roswell Park Memorial Institute (RPMI) 1640 and allowed to adhere to plates at a final concentration of 5 × 10^6^/mL. After 37 °C overnight, nonadherent cells were gently removed. The resulting adherent cells were cultured in medium supplemented with GM-CSF (100 ng/mL, Peprotech) and IL-4 (100 ng/mL, Peprotech) in 5% CO_2_ at 37 °C. Every 2 d, one-half of the medium was replaced by fresh medium containing double concentration (DC) of GM-CSF and IL-4 as indicated above. After 5 d of culture, 10 ng/mL recombinant human tumor necrosis factor (TNF-α, Peprotech) was added to the medium to induce phenotypic and functional maturation. Then, 48 h later, DCs were pulsed with 20 μg/mL SSP-1 peptide in the presence of 3μg/mL β-microglobulin (Sino Biological) at 37 °C for 3 h before use. PBLs (2 × 10^6^) were cocultured with 2 × 10^5^ peptide-pulsed autologous DCs in a 24-well plate in the presence of 10 ng/mL recombinant human interleukin-2 (IL-2; Peprotech). The next day, recombinant human IL-10 (Peprotech) was added to the culture medium to give a final concentration of 10 ng/mL. After 7 d, lymphocytes were restimulated with peptide-pulsed autologous DCs in medium containing 10 ng/mL IL-2. Lymphocytes were restimulated each week in the same manner. At 7 d after the fourth round of restimulation, cells were harvested and CD8^+^ cells were purified by microbeads (BD Bioscience) tested by cytotoxicity assay and tetramer staining.

### IFN-γ ELISpot.

The PBMCs derived from recovered SARS-CoV-2–infected patients were isolated from peripheral blood by Ficoll-Hypaque gradient separation. PBMCs (1 × 10^6^/mL) were cultured with S peptides pool (GenScript). One-half of the medium was changed at day 3 with supplementation of IL-2 at 10 ng/mL At day 7, IFN-γ–secreting T cells were detected by Human IFN-γ ELISpot assay kits (DKW22-1000-096s; Dakewe) according to the manufacturer’s protocol. PBMCs were plated in duplicate at 4 × 10^5^ per well and then incubated 24 h. Spots were then counted using an S6 μLtra immunoscan reader (Cellular Technology Ltd.), and the number of IFN-γ–positive T cells was calculated by ImmunoSpot 5.1.34 software (Cellular Technology Ltd.) The number of spots was converted into the number of spots per million cells and the mean of duplicate wells plotted.

### Generation of Specificity Tetramer Using Peptide Exchange.

The peptide exchange experiment was performed with QuickSwitch Quant HLA-A*02:01 Tetramer Kit-PE (MBL) according to the manufacturer’s instructions. Briefly, we dissolved each lyophilized peptide (SSp-1) in DMSO at a stock concentration of 10 mM. A total of 50 μL of QuickSwitchTM Tetramer was pipetted into a microtube, and 1 μL of target peptide was added and mixed gently with pipetting. Then, 1 μL of peptide exchange factor was added and mixed gently with pipetting. The samples were incubated at least for 4 h at room temperature protected from light.

### CTL Killing Assay.

1) For the killing assay for the CTLs generated from healthy donors, CTLs were isolated and counted. A total of 5 × 10^5^ HEK293T cells transfected with either 3.1-GFP, SARS-CoV ORF8a-GFP, or SARS-CoV-2 ORF8-GFP were loaded with 20 μg/mL SSP-1 peptides or HIV-gag peptides (SL9) at 37 °C for 1 h (42). The CD8^+^ T cells were cocultured with target cells at the indicated ratios overnight.
2) For restimulation of CD8 T cells isolated from the recovered SARS-CoV-2–infected patients, the PBMCs were cultured with the synthetic peptide mixture of SARS-CoV-2 at a concentration of 1μg/mL or DMSO in RPMI medium 1640 containing 10% fetal calf serum and 20 U/mL recombinant human IL-2 (Peprotech) for 7 d. Then, CTLs from recovered SARS-CoV-2–infected patients were isolated and counted. A total of 5 × 10^5^ HEK293T cells transfected with either 3.1-GFP or ORF8-GFP were loaded with 20 μg/mL synthetic peptide mixture of SARS-CoV-2 or HIV-gag peptides (SL9) at 37 °C for 1 h (42). The CD8^+^ T cells were cocultured with target cells.

Afterward, cells were labeled with the fixable viability dye eFluor 780 (eBioscience) and analyzed by flow cytometry. For determination of antigen, nontarget ratio cell counts of dead SARS-CoV-2 peptides–loaded GFP^+^ cells were divided by the counts for dead HIV-gag peptides–loaded GFP^+^ cells (52).

### Killing Assay for Authentic SARS-CoV-2–Infected 293T Cells.

HEK293T cells were transfected with pcCMV-ACE2-Flag with and without pORF8-scFv-VIF-1. After 24 h, cells were washed with PBS and infected with authentic SARS-CoV-2 at MOI = 1 for 1 h at 37 °C. Then, cells were washed with PBS and replaced with DMEM (2% FBS). At 24 h after infection, these target cells were cocultured with CD8^+^ T cells generated from healthy donors at the indicated ratios overnight. Afterwards, cells were stained with propidium iodine (Biolegend) and CD8 then analyzed by flow cytometry. For determination of antigen, nontarget ratio cell counts of dead SARS-CoV-2–infected cells were divided by the counts for dead uninfected cells.

### Statistical Analysis.

Differences between two or more groups were analyzed by Student’s *t* test or one-way ANOVA followed by Tukey's test. Statistical significance performed using GraphPad Prism 6. Flow cytometry results were analyzed using FlowJo software (Tree Star Inc.) *P* < 0.05 indicates a statistically significance difference.

## Supplementary Material

Supplementary File

## Data Availability

All study data are included in the article and/or *SI Appendix*.
