# Supplementary material for: The ORF8 protein of SARS-CoV-2 mediates immune evasion through down-regulating MHC-Ι
Source: Proc Natl Acad Sci U S A. 2021 May 21;118(23):e2024202118. doi: 10.1073/pnas.2024202118 (PMC8201919; doi:10.1073/pnas.2024202118)
Supplement: Supplementary File [file pnas.2024202118.sapp.pdf]

Supplemental Materials

Supplement Table 1. Sequence of ORF8-scFv-1 and ORF8-scFv-2.

ORF8-scFv-1:

ATGGAAATCGTGCTGACACAGAGCCCCGGCACACTGTCACTTTCTCCAGGCGAGAC
AGCCATCATCAGCTGTCAGGCCCAGATCATCACCCACTGCTGCTGGTATCAGCAGAG
GCCTGGACAGGCCCCTAGACTGGTCATCTACCCTGAGATCTGGTCCAAGAGAGGCA
TCCCCGACAGATTCAGCGGCTCTAGATGGGGCCCTGACTACAACCTGACCATCAGC
AACCTGGAAAGCGGCGACTTCGGCGTGTACTACTGCATGAGCGAGCCCAGATTTGG
CCAGGGCACAAAGGTGCAGGTCGACATCAAGAGATCTGGCGGCGGAGGATCTTCT
GGCGGAGGTGGAAGTAGTGGCGGAGGCGGATCTCAGGTTCAGCTGGTTCAAAGCG
GCGGACAGATGAAGAAACCCGGCGAGAGCATGCGGATCAGCTGCAGAGCCAGCAG
ATGCCACATCAGAGTGATGGACCACATCGGCTTCGTGTGGCCTCCAGAGAAGGGAC
TGTCTGGCTGGGATGGACAGTCTCTCGGCGGATCTAGAGCACCTCCATTCGACAGC
ACCCACGTGGAACAGCCCCAGCTGGAAACCTTTATTCCCACACAGCCCTTTTGGAG
CTGCGCCAGACAGCAGCAGACCACCAGACCTAGCACATTCGTGCTGGGCACCCTGT
ACAACACCAAGATGGCCTACTGTGCCAGATGGGCTGGCGCCCCTAGATCTTCTTCTC
AC

ORF8-scFv-2:

ATGGAAATCGTGCTGACACAGAGCCCCGGCACACTGTCACTTTCTCCAGGCGAGAC
AGCCATCATCAGCTGTCAGGCCCAGATCATCACCCACTGCTGCTGGTATCAGCAGAG
GCCTGGACAGGCCCCTAGACTGGTCATCTACCCTGAGATCTGGTCCAAGAGAGGCA
TCCCCGACAGATTCAGCGGCTCTAGATGGGGCCCTGACTACAACCTGACCATCAGC
AACCTGGAAAGCGGCGACTTCGGCGTGTACTACTGCATGAGCGAGCCCAGATTTGG
CCAGGGCACAAAGGTGCAGGTCGACATCAAGAGATCTGGCGGCGGAGGATCTTCT
GGCGGAGGTGGAAGTAGTGGCGGAGGCGGATCTCAGGTTCAGCTGGTTCAAAGCG
GCGGACAGATGAAGAAACCCGGCGAGAGCATGCGGATCAGCTGCAGAGCCTCTAG
ATGCCAGCACACCAGATACGGCCCCTACTGGATTAGACTGGCCCCTGGCAAAAGAC
CCGAGTGGATGGGATGGCTGAAGCCCAGAGGAATTGCCAGCGCCGCCATCCAGTTT
CACCCCTGTAGAGTGACCATGACCAGGGACGTGTACAGCGATACCGCCTTCCTGGA
ACTGAGAAGCCTGACCGTGGATGATACCGCCGTGTACTTCTGTACCCGGGACCTGAT
CCAGCACGAGGACGGCATCCTGTGTTCTGTCGGCAGAGGCACACCTGTGATCGTGT
CTAGT

Supplementary information

Antibodies

Reagent or Resource	Source	Identifier
HLA-A2-APC (BB7.2)	BD Biosciences	Cat# 561341
HLA-A,B,C-APC (W6/32)	Biolegend	Cat# 311410
HLA-A2-APC (BB7.2)	Biolegend	Cat# 343308
β2-microglobulin-PE (2M2)	Biolegend	Cat# 316306
Fixable Viability Dye eFluor 780	Thermo Scientific	Cat# 65-0865-14
CD8-BV421 (RPA-T8)	BD Biosciences	Cat# 562428
MHC class I Rabbit mAb	Abclonal	Cat# A8754
HLA Class I ABC Antibody	Proteintech	Cat# 15240-1-AP
HLA Class I Antibody	Immunoway	Cat# YT5837
Calreticulin (D3E6) Rabbit mAb	Cell Signaling Technology	Cat# 12238T
LAMP1 (D2D11) Rabbit mAb	Cell Signaling Technology	Cat# 9091T
LC3A/B (D3U4C) Rabbit mAb	Cell Signaling Technology	Cat# 12741S
GM130 (D6B1) Rabbit mAb	Cell Signaling Technology	Cat# 12480S
Rab5 (C8B1) Rabbit mAb	Cell Signaling Technology	Cat# 3547T
HLA A2 antibody	Abcam	Cat# ab168405

Chemicals, Peptides, and Recombinant Proteins

Reagent or Resource	Source	Identifier
HLA-A cDNA ORF Clone, Human, C- Flag tag	sinobiological	Cat# HG13263-CF
Calreticulin cDNA ORF Clone, Human, C-HA tag	sinobiological	Cat# HG13539-CY
RAB5 cDNA ORF Clone	sinobiological	Cat# HG14013-UT
RAB7A cDNA ORF Clone, C- Flag tag	sinobiological	Cat# HG16168-NF
Beta-2 microglobulin Protein, Human, Recombinant (His Tag)	sinobiological	Cat# 11976-H08H
Beclin 1 cDNA ORF Clone, Human, C-GFP tag	sinobiological	Cat# HG11162-ACG
DSP	Thermo Scientific	Cat# 22586
Pepstatin	TargetMol	Cat# T3695
DBeQ	TargetMol	Cat# T1969

MG-132	TargetMol	Cat# T2154
Aloxistatin (E64d)	TargetMol	Cat# T6040
Bafilomycin A1	Selleck	Cat# S1413
Recombinant Human GM-CSF	Peprotech	Cat# 300-03
Recombinant Human IL-4	Peprotech	Cat# 200-04
Recombinant Human TNF-α	Peprotech	Cat# 300-01A
Recombinant Human IL-2	Peprotech	Cat# 200-02
Recombinant Human IL-10	Peprotech	Cat# 200-10

Critical Commercial Assays

**Reagent or Resource**	**Source**	**Identifier**
Sybr Green PCR Master Mix	Thermo Scientific	Cat# 4309155
QuickSwitch Quant HLA-A*02:01 Tetramer Kit-PE	MBL	Cat# TB-7300-K1
Human CD8 T Lymphocyte Enrichment Set-DM	BD Biosciences	Cat# 557941
Lipofectamin RNAiMAX Transfection Reagent	Thermo Scientific	Cat# 13778150
Lipofectamin 2000 Transfection Reagent	Thermo Scientific	Cat# 11668019
Lysosome Isolation Kit	Millipore Sigma	Cat# LYSISO1
ProteoSilver™ Silver Stain Kit	Millipore Sigma	Cat# PROTSIL1
Human IFN-γ ELISpot assay kits	Dakewe	Cat# DKW22-1000-096s
FITC Annexin V Apoptosis Detection Kit with PI	Biolegend	Cat# 640914

Supplement Fig. 1

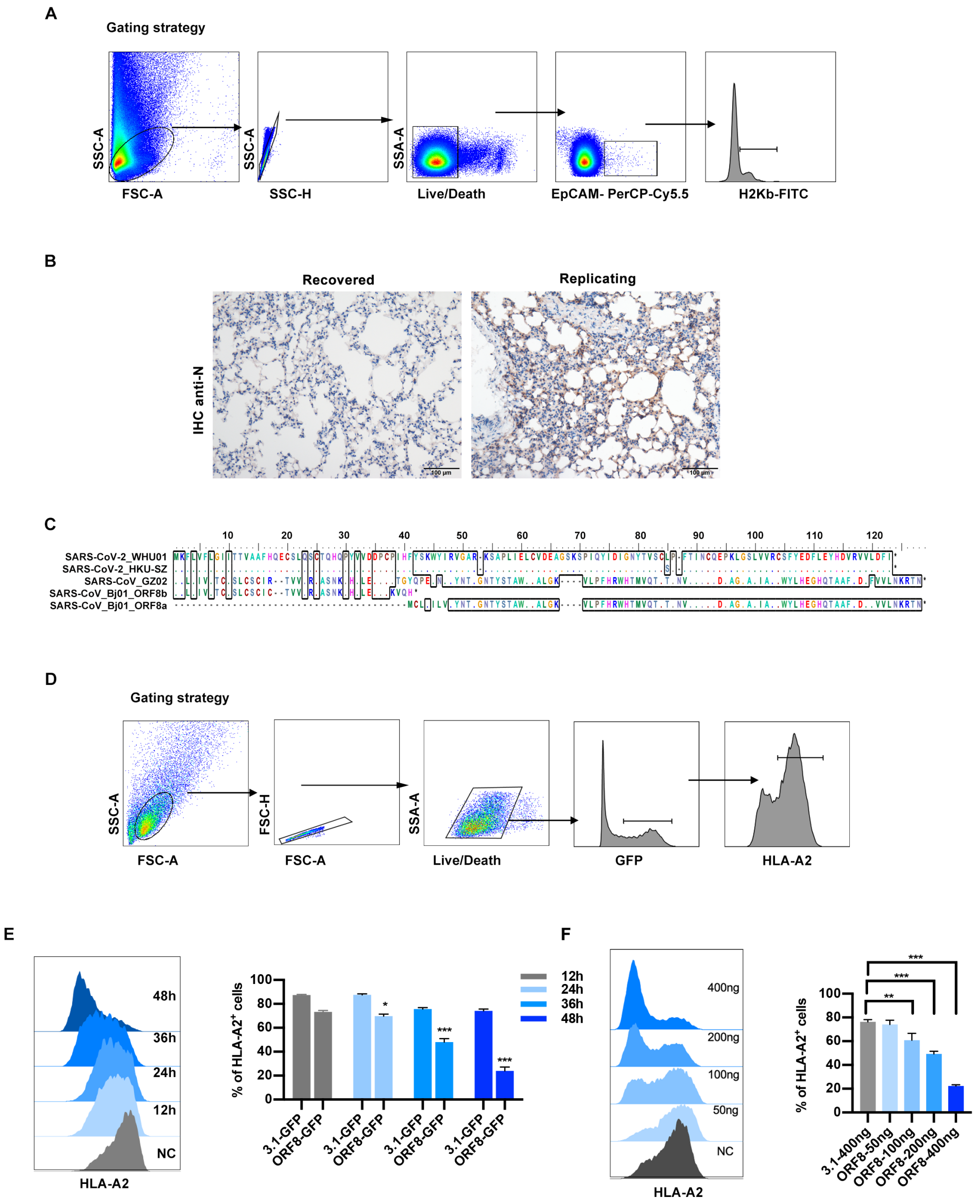

Supplement Fig. 1 Surface expression of MHC-I is downregulated in ORF8-expressing cells.

(A) Gating strategy for FACS analysis of H2Kb expression on lung epithelial cells of mice. **(B)** hACE2 mice were intranasal infected with 4×10^3 PFU (recovered), 4×10^4 PFU (replicating) SARS-CoV-2 virus, or uninfected as control. At day 6 post infection, lung tissues were collected for IHC staining. **(C)** The multiple alignment was created based on the amino acid sequences of the encoded protein of orf8 gene, including 1 from SARS-CoV-2_WHU01, 1 from SARS-CoV-2_HKU-SZ with L84S

mutation, 1 from SARS-CoV_GZ02, and 1 from SARS-CoV_BJ01 orf8a, 1 from SARS-CoV_BJ01 orf8b. The similarity shading with the color was referred to the chemistry of each amino acid at that position. **(D)** Gating strategy for FACS analysis of MHC-I expression on HEK293T cells used in this study. **(E)** GFP, or ORF8-GFP expressing plasmids were transfected into HEK293T cells, respectively. Cells were harvested for flow cytometry analysis at the indicated time points. Frequency of HLA-A2^{+} cells (gated on GFP^{+} cells) were shown (n=5). **(F)** Different doses of GFP, or ORF8-GFP expressing plasmids were transfected into HEK293T cells, respectively. After 48 hours cells were harvested for flow cytometry. Frequency of HLA-A2^{+} cells (gated on GFP^{+} cells) were shown (n=5). Data were shown as mean ± SD (error bars). t test and one way ANOVA was used. $P < 0.05$ indicates statistically significance difference. * indicates $P < 0.05$; ** indicates $P < 0.01$; *** indicates $P < 0.001$.

Supplement Fig.2

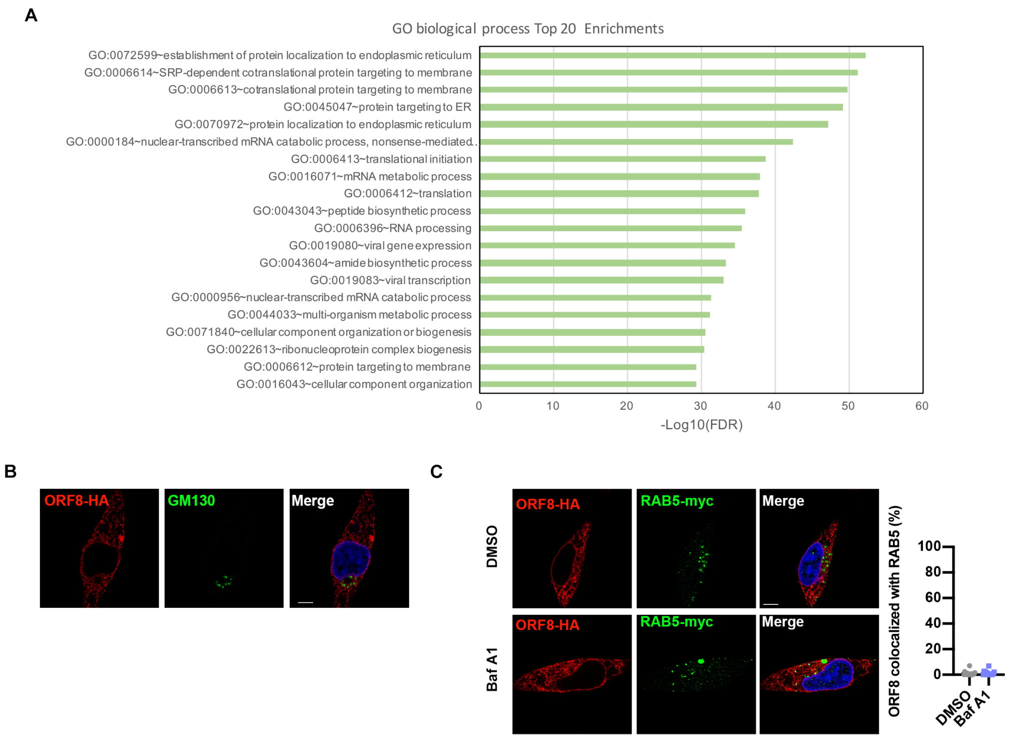

Supplement Fig.2 ORF8 interacting with endoplasmic reticulum (ER). (A) The top 20 significant enrichments of the gene ontology (GO) biological process terms. Transformed false discovery rate (FDR) was indicated at the X-axis. **(B)** Localization of SARS-CoV-2 ORF8 (red) relative to GM130 (green, the top panel). ORF8-HA expressing plasmid was transfected into HEK293T cells. At 24 hours after transfection, co-localization was visualized by confocal microscopy. Scale bars, 5μm. **(C)** Localization of SARS-CoV-2 ORF8 (red) relative to RAB5 (green). ORF8-HA expressing plasmid together with RAB5-myc expressing plasmid were transfected into HEK293T cells. At 24 hours after transfection, co-localization was visualized by confocal microscopy. Scale bars, 5μm.

Supplement Fig.3

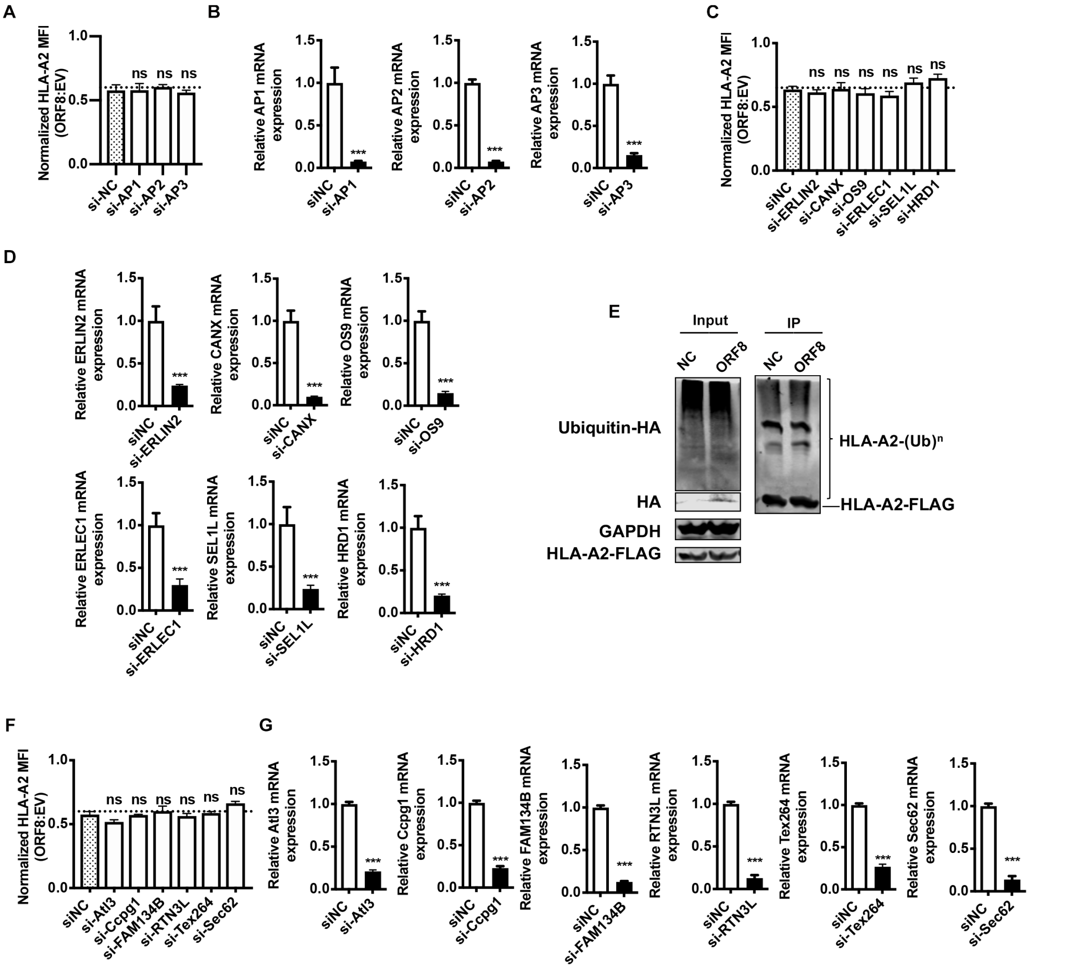

Supplement Fig.3 ORF8 mediates MHC-I trafficking from ER to lysosome for degradation. (A) GFP (EV) or ORF8-GFP expressing plasmids, and the indicated siRNAs were transfected into HEK293T cells. MFI of HLA-A2 (gated on GFP^+ cells) was normalized to GFP group (n=5). **(B)** The knockdown efficiency of indicated siRNAs (n=3). **(C)** GFP (EV) or ORF8-GFP expressing plasmids, and the indicated siRNAs were transfected into HEK293T cells. MFI of HLA-A2 (gated on GFP^+ cells) was normalized to GFP group (n=5). **(D)** The knockdown efficiency of indicated siRNAs (n=3). **(E)** HLA-A2-FLAG and ubiquitin-HA expressing plasmids, in combination with ORF8-HA expressing plasmid or empty vector were transfected into HEK293T cells. Cells were treated with MG132 (10μM) for 12 h before harvest. HLA-A2 ubiquitination was analyzed by Co-IP with anti-Flag-tag beads and followed by western botting. **(F)** GFP (EV) or ORF8-GFP expressing plasmid, and the indicated siRNAs were transfected into HEK293T cells. MFI of HLA-A2 (gated on GFP^+ cells) was normalized to 3.1-GFP (n=5). (G) The knockdown efficiency of indicated siRNAs (n=3). Data were shown as mean ± SEM (error bars). t test was used. $P < 0.05$ indicates statistically significance difference. *** indicates $P < 0.001$.

Supplement Fig.4

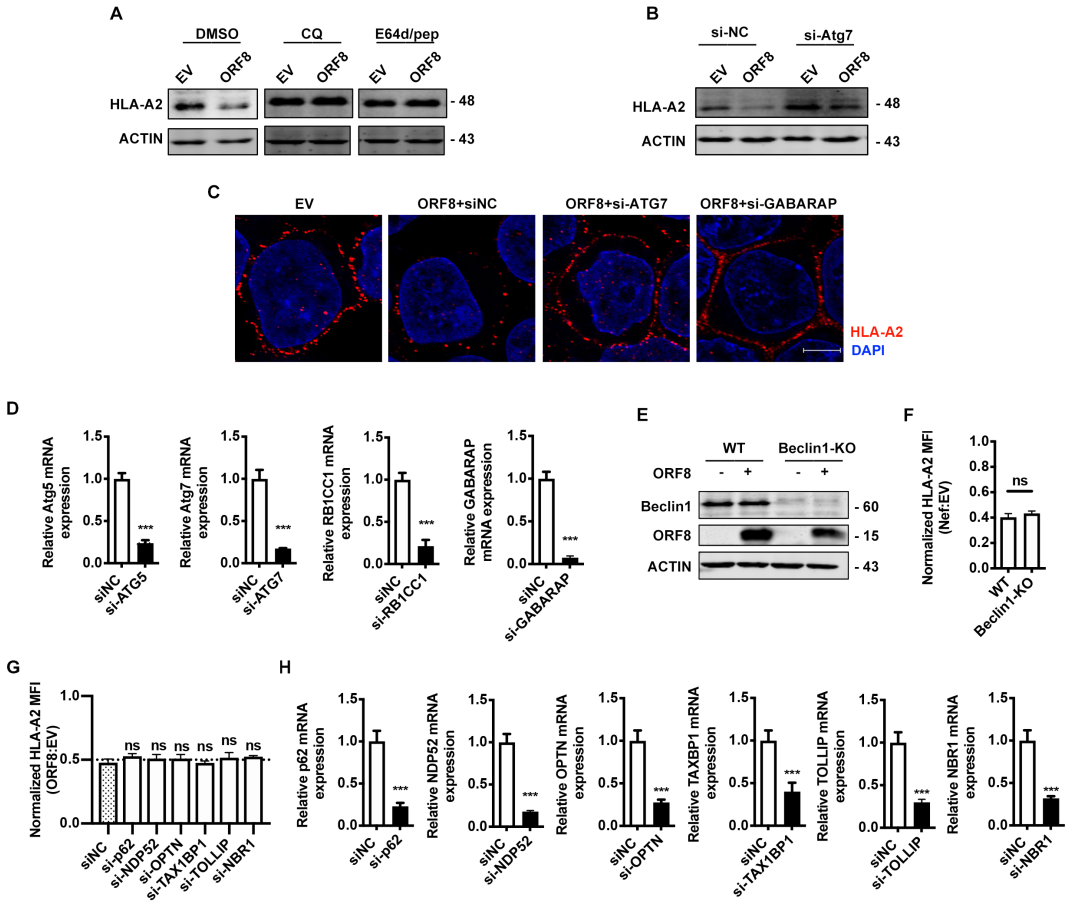

Supplement Fig.4 ORF8 mediates MHC-I degradation though autophagy pathway.

(A) GFP (EV) or ORF8-GFP expressing plasmid were transfected into HEK293T cells. Before harvest, cells were treated with chloroquine (CQ) (50 μM), E64d (10ug/mL), or pepstatin A (pep) (10ug/mL) for 6 hours. The total HLA-A2 protein expression was analyzed by western blotting (n=3). **(B)** The GFP (EV) or ORF8-GFP expressing plasmids, and the indicated siRNAs were transfected into HEK293T cells. The total HLA-A2 protein expression was analyzed by western blotting (n=3). **(C)** The ORF8-HA expressing plasmid and the indicated siRNAs were transfected into HEK293T cells. At 48 hours after transfection, HLA-A2 localization was visualized by confocal microscopy. **(D)** The knockdown efficiency of indicated siRNAs (n=3). **(E)** GFP (EV) or ORF8-GFP expressing plasmids were transfected into HEK293T cells (WT), or Beclin1 knockout HEK293T cells. At 48 hours after transfection, cells were collected for western blotting (n=3). **(F)** GFP (EV) or Nef-GFP expressing plasmids were transfected into HEK293T cells (WT), or Beclin1 knockout HEK293T cells. MFI of HLA-A2 (gated on GFP^+ cells) was normalized to GFP group (n=5). **(G)** The GFP (EV) or ORF8-GFP expressing plasmids, in combination with the indicated siRNAs were transfected into HEK293T cells. MFI of HLA-A2 (gated on GFP^+ cells) was normalized to GFP group (n=5). **(H)** The knockdown efficiency of indicated siRNAs (n=3). t test was used. *** indicates P < 0.001.
